# Dicer functions as an antiviral system against human adenoviruses *via* cleavage of adenovirus-encoded noncoding RNA

**DOI:** 10.1038/srep27598

**Published:** 2016-06-07

**Authors:** Mitsuhiro Machitani, Fuminori Sakurai, Keisaku Wakabayashi, Kyoko Tomita, Masashi Tachibana, Hiroyuki Mizuguchi

**Affiliations:** 1 Laboratory of Biochemistry and Molecular Biology, Graduate School of Pharmaceutical Sciences, Osaka University, 1-6 Yamadaoka, Suita, Osaka 565-0871, Japan; 2 Laboratory of Regulatory Sciences for Oligonucleotide Therapeutics, Clinical Drug Development Unit, Graduate School of Pharmaceutical Sciences, Osaka University, 1-6 Yamadaoka, Suita, Osaka, 565-0871, Japan; 3 Laboratory of Hepatocyte Regulation, National Institute of Biomedical Innovation, Health and Nutrition, 7-6-8 Saito, Asagi, Ibaraki, Osaka 567-0085, Japan; 4 iPS Cell-Based Research Project on Hepatic Toxicity and Metabolism, Graduate School of Pharmaceutical Sciences, Osaka University, 1-6 Yamadaoka, Suita, Osaka 565-0871, Japan; 5 Global Center for Advanced Medical Engineering and Informatics, Osaka University, 2-2 Yamadaoka, Suita, Osaka 565-0871, Japan; 6 Graduate School of Medicine, Osaka University, 2-2 Yamadaoka, Suita, Osaka 565-0871, Japan

## Abstract

In various organisms, including nematodes and plants, RNA interference (RNAi) is a defense system against virus infection; however, it is unclear whether RNAi functions as an antivirus system in mammalian cells. Rather, a number of DNA viruses, including herpesviruses, utilize post-transcriptional silencing systems for their survival. Here we show that Dicer efficiently suppresses the replication of adenovirus (Ad) *via* cleavage of Ad-encoding small RNAs (VA-RNAs), which efficiently promote Ad replication *via* the inhibition of eIF2α phosphorylation, to viral microRNAs (mivaRNAs). The Dicer knockdown significantly increases the copy numbers of VA-RNAs, leading to the efficient inhibition of eIF2α phosphorylation and the subsequent promotion of Ad replication. Conversely, overexpression of Dicer significantly inhibits Ad replication. Transfection with mivaRNA does not affect eIF2α phosphorylation or Ad replication. These results indicate that Dicer-mediated processing of VA-RNAs leads to loss of activity of VA-RNAs for enhancement of Ad replication and that Dicer functions as a defence system against Ad in mammalian cells.

RNA interference (RNAi) is an evolutionarily conserved system in almost all eukaryotes. RNAi works as an antiviral system in plants, nematodes, and insects. Double-stranded RNAs (dsRNAs) derived from invading viruses are cleaved by Dicer into small interfering RNAs (siRNAs). RNA-induced silencing complexes (RISCs) containing virus-derived siRNAs cleave viral RNA genomes, leading to the suppression of virus replication^1^,^2^. On the other hand, it has remained to be clarified whether RNAi functions as an antiviral system in mammalian cells, although RNAi is functional and is utilized for not only basic research but also therapeutic applications in mammals. Recently, two studies demonstrated that the RNA genomes of encephalomyocarditis virus (EMCV) and nodamura virus were cleaved by RNAi in restricted mouse cell lines^3^,^4^.

On the other hand, it remains to be revealed whether the RNAi system acts as a defense against DNA viruses in mammalian cells. Rather, a number of DNA viruses utilize post-transcriptional silencing systems for their survival. Several DNA virus families encode microRNAs (miRNAs) in their genomes^1^,^5^. Viral miRNAs are processed similarly to miRNAs in eukaryotic cells. Viral pre-miRNAs are transported from the nucleus into the cytosol following transcription from the DNA virus genome, followed by Dicer-mediated cleavage and subsequent incorporation into RISC. RISC that contains viral miRNAs suppresses the expression of not only host genes but also virus genes for virus survival^5^. For example, miR-LAT, an miRNA encoded by herpes simplex virus type 1, inhibits the expression of apoptosis signaling mediators, transforming growth factor (TGF)-β and SMAD3, *via* post-transcriptional silencing, leading to the establishment of latent infection^6^.

The adenovirus (Ad) genome also encodes two noncoding small RNAs, VA-RNA I (a major species) and VA-RNA II (a minor species), which are approximately 160 nucleotide-long noncoding RNAs that are transcribed by RNA polymerase III^7^,^8^. VA-RNA I is rapidly transcribed after infection and accumulates to very high levels, 10^8^ molecules per cell. In previous studies, an Ad mutant lacking VA-RNA expression, Sub720, grew 60-fold less efficiently^9^,^10^,^11^,^12^, suggesting that VA-RNAs were crucial for efficient amplification of Ad. The molecular mechanism of VA-RNA I in supporting Ad amplification works, at least in part, by antagonizing the antiviral action associated with the Ad-induced activation of dsRNA-dependent protein kinase (PKR)^12^. After infection with Ads, VA-RNA I is transcribed from the Ad genome in the infected cells. Subsequently, the VA-RNA I binds to PKR with high affinity and blocks PKR activation. This blocking leads to the inhibition of eIF2α phosphorylation, leading to the maintenance of viral protein synthesis and the enhancement of Ad amplification^12^,^13^. VA-RNA II also supports Ad replication^8^, although VA-RNA II has not been demonstrated to inhibit PKR activation^14^. VA-RNA I and II have a similar stem-loop structure *via* an intramolecular hydrogen bond. The apical stem and central domain in VA-RNA I play an important role in blocking PKR activation^8^,^15^.

Recently, several studies have demonstrated that VA-RNAs were also processed in a manner similar to miRNAs^16^,^17^,^18^,^19^,^20^,^21^, resulting in the production of VA-RNA-derived miRNAs (mivaRNAs), which are approximately 22-nt long. mivaRNAs are incorporated into RISC and exhibit post-transcriptional silencing in a manner similar to miRNAs. However, it remains to be clarified whether Dicer-mediated processing of VA-RNAs and the following production of truncated VA-RNAs (VAΔmivas) and mivaRNAs are crucial for Ad replication.

In the present study, we demonstrate that Dicer-mediated processing of VA-RNAs negatively regulates Ad replication and that a knockdown of Dicer leads to the promotion of Ad replication, indicating that Dicer acts as an antiviral system against Ad *via* cleavage of VA-RNAs.

## Material and Methods

### Cells and reagents

HeLa (a human epithelial carcinoma cell line), HEK293 (a transformed embryonic kidney cell line), 293T (a transformed embryonic kidney cell line expressing SV40 large T antigen), SK HEP-1 (a human hepatoma cell line), and HepG2 (a human hepatoma cell line) cells were cultured in Dulbecco’s modified Eagle’s medium (DMEM) supplemented with 10% fetal bovine serum (FBS), streptomycin (100 μg ml^−1^), and penicillin (100 U ml^−1^). In the experiments shown in Supplementary Figure S9, HeLa cells were cultured in DMEM supplemented with 2%, 5%, or 10% FBS and the antibiotics. H1299 (a non-small cell lung carcinoma cell line), MCF-7 (a breast cancer cell line), and adriamycin-resistant MCF-7 (MCF-7/ADR) cells (kindly provided by Dr. T. Ochiya, National Cancer Center Research Institute, Tokyo, Japan) were cultured in RPMI1640 supplemented with 10% FBS, streptomycin (100 μg ml^−1^), and penicillin (100 U ml^−1^). Control siRNA was purchased from Qiagen (Allstars Negative Control siRNA; Qiagen, Hilden, Germany). siRNAs against Dicer (siDicer#2) and Argonaute 2 (Ago2) (siAgo2) were purchased from Thermo Fisher Scientific (Darmacon SMARTpool; Thermo Fisher Scientific, Lafayette, CO). siDicer#1 and siRNAs against PKR (siPKR) were obtained from Gene Design (Osaka, Japan). The target sequences of siDicer#1, and siPKR were 5′-gaatcagcctcgcaacaaa-3′ and 5′-ggtgaaggtagatcaaaga-3′, respectively.

### Plasmids

The control plasmid pAdVAntage-ΔNaeI with deleted VA-RNA expression was previously constructed using pAdVAntage^22^. A VA-RNAI-expressing plasmid, pVAI, and a VA-RNAII-expressing plasmid, pVAII, were constructed as follows: pAdVAntage (Promega, Madison, WI), which encodes VA-RNA I and II under the control of an endogenous RNA polymerase III promoter, was digested with *Apa*I/*Nhe*I and *Eco*RV/*Nhe*I, respectively. The resulting fragments were self-ligated after the sticky end was converted to a blunt end, resulting in pVAI and pVAII, respectively. The VA-RNA expression profiles of these plasmids were confirmed by quantitative RT-PCR analysis (Supplementary Figure S1).

pAdHM41-E3(+)-GFP, the Ad vector plasmid carrying a CMV promoter-driven green fluorescent protein (GFP) expression cassette, was previously constructed^23^. The Ad vector plasmid encoding wt-VAI and lacking VA-RNA II expression, pAdΔVR7, and the Ad vector plasmid encoding mut-VAI and lacking VA-RNA II expression, pAdΔVR8, were constructed by homologous recombination in *E. coli* as follows: briefly, a fragment of Ad genome (bp 1–21562) was cloned into pGEM7-Zf(+) (Promega) after deletion of the transcriptional control element of VA-RNA II expression (bp 10925–10944), resulting in pGEM7.5-Ad5. A fragment encoding the mut-VAI gene, which was synthesized (Greiner Bio-One, Tokyo, Japan), was inserted into the *Bgl*II/*Nhe*I site of pGEM7.5-Ad4^24^, resulting in pGEM7.5-Ad6. *E. coli* BJ5183 (recBC and sbcBC) (Agilent Technology, Palo Alto, CA) was co-transformed with the *Sbf* I/*Xmn*I fragment of pGEM7.5-Ad5 or pGEM7.5-Ad6 and *Pme*I-digested pAdHM41-E3(+)^23^ by electroporation, resulting in pAdΔVR7 or pAdΔVR8, respectively. The sequences of VA-RNA genes in pAdΔVR7 or pAdΔVR8 were verified by sequence analysis. pHMCMV-GFP1^25^ containing the CMV promoter-driven GFP gene was digested with I-*Ceu*I/PI-*Sce*I and then ligated with I-*Ceu*I/PI-*Sce*I-digested pAdΔVR7 or pAdΔVR8, resulting in pAdΔVR7-GFP or pAdΔVR8-GFP, respectively.

The human Dicer gene was amplified by PCR using cDNA prepared from HEK293 cells and cloned. Further details on the construction methods are available upon request.

### Viruses

Wild-type Ad serotype 5 (WT-Ad), Ad31, Ad11, Ad35, and Ad4 were obtained from American Type Culture Collection (ATCC). These Ads were propagated in HEK293 cells. Sub720^22^, a mutant Ad serotype 5 lacking the expression of both VA-RNAI and II, was propagated in 293T cells.

Recombinant Ads were prepared as follows. *Pac*I-digested pAdHM41-E3(+)-GFP, pAdΔVR7-GFP, or pAdΔVR8-GFP were each transfected into HEK293 cells using Lipofectamine 2000 (Invitrogen, Carlsbad, CA), resulting in AdV, AdV-VAI, and AdV-mutVAI, respectively. These recombinant Ads were amplified and purified by two rounds of cesium chloride-gradient ultracentrifugation, dialyzed, and stored at −80 °C^26^. Determination of Infectious unit (IFU) titers was accomplished using an Adeno-X Rapid Titer Kit (Clontech, Mountain View, CA).

### Stable cell transformants inducibly expressing shDicer

HeLa, SK HEP-1, HepG2, and H1299 cells were transduced with a lentivirus vector inducibly expressing shDicer and constitutively expressing tetracycline repressor and monomeric red fluorescent protein (mRFP1) as a single transcript linked with a 2A-self-cleaving peptide (LV-H1tetO-shDicer-ETR) (Supplementary Figure S2) (see Supplementary Information). The resulting cells were designated HeLa-shDicer, SK HEP-1-shDicer, HepG2-shDicer, and H1299-shDicer cells, respectively.

### Northern blotting analysis

Total RNA was extracted from the cells with ISOGEN (Nippon Gene, Tokyo, Japan). Ten micrograms of total RNA per lane was loaded onto 15% polyacrylamide denaturing gel. After electrophoresis, bands of RNA were transferred to Hybond-N+ membranes (Roche, Mannheim, Germany). The membranes were then probed with ^32^P-labeled synthetic oligonucleotides that were complementary to the sequence of VA-RNA I, II, or human U6 small nuclear RNA (VA-RNA I: 5′-aggagcgctcccccgttgt-3′; VA-RNA II: 5′-gggctcgtccctgtttcc-3′; U6: 5′-tgctaatcttctctgtatcgt-3′).

### Western blotting analysis

Western blotting assay was performed as previously described^27^. Briefly, whole-cell extracts were prepared and electrophoresed on 10% sodium dodecyl sulfate (SDS)–polyacrylamide gels under reducing conditions, followed by electrotransfer to PVDF membranes (Millipore, Bedford, MA). After blocking with 5% skim milk prepared in TBS-T (tween-20, 0.1%), the membrane was incubated with primary antibodies (Supplementary Table S3), followed by incubation in the presence of horseradish peroxidase (HRP)-labeled anti-mouse or anti-rabbit IgG antibody (Cell Signaling Technology, Danvers, MA). The protein bands of the membrane were visualized with a chemiluminescence kit (ECL Plus Western blotting detection system; Amersham Biosciences, Piscataway, NJ). The intensity of protein bands was quantified by Image J software.

### Real-time RT-PCR analysis

Real-time RT-PCR analysis was performed as previously described^28^. Complementary DNA was synthesized using 500 ng of total RNA with a Superscript VILO cDNA synthesis kit (Invitrogen). Real-time RT-PCR analysis was performed with THUNDERBIRD SYBR qPCR Mix (TOYOBO, Osaka, Japan) using StepOnePlus real-time PCR systems (Applied Biosystems, Foster City, CA). The primer sequences used in this study are described in Supplementary Table S2.

### Determination of Ad genome copy numbers

Total DNA, including Ad genomic DNA, was isolated from the cells infected with Ads using a DNeasy Blood & Tissue Kit (Qiagen). After isolation, Ad genome copy numbers were quantified using StepOnePlus real-time PCR systems (Applied Biosystems) as previously described^29^. The primer and probe sequences used in this study are described in Supplementary Table S2.

### Preparation of Dicer-processed VA-RNA I

VAΔmivaI, a truncated VA-RNA I lacking the terminal stem, was constructed as follows. First, pUC19-VAΔmivaI, which includes a VAΔmivaI sequence, was obtained from Greiner Bio-One. Next, a fragment encoding VAΔmivaI was amplified by PCR using primers T7-VAΔmivaI-F and T7-VAΔmivaI-R, and using pUC19-VAΔmivaI as a template. A T7 promoter sequence was included in T7-VAΔmivaI-F to allow *in vitro* transcription using the MEGAshortscript Kit (Ambion Austin, TX). The *in vitro* transcribed VAΔmivaI was electrophoresed on polyacrylamide and TBE-Urea gels under nondenaturing and denaturing conditions, respectively, to confirm the size of VAΔmivaI (Supplementary Figure S3). Chemically synthesized mivaRNAI was obtained from Qiagen. We confirmed by northern blotting analysis that the chemically synthesized mivaRNAI and the endogenous mivaRNAI, which was isolated from WT-Ad–infected cells, were the same size (Supplementary Figure S7).

### Reporter plasmids and reporter assay

Reporter plasmids, psiCHECK-2-mivaRNAIT and -mut-mivaRNAIT, containing two copies of a sequence complementary to mivaRNAI and mut-mivaRNAI, respectively, in the 3′-UTR of the RLuc gene, were constructed as follows. An *Xho*I/*Not*I fragment of psiCHECK-2 (Promega) was ligated with oligonucleotides encoding sequences complementary to mivaRNAI, mivaRNAIT-S, and mivaRNAIT-AS, resulting in psiCHECK-2-mivaRNAIT. psiCHECK-2-mut-mivaRNAIT was similarly constructed using the corresponding oligonucleotides. The sequences of the oligonucleotides are shown in Supplementary Table S2.

HEK293 cells were transfected with the reporter plasmids, followed by transduction with AdV, AdV-VAI, or AdV-mutVAI. After 48 h incubation, luciferase activity in the cells was determined using the Dual Luciferase Reporter Assay System (Promega).

### Infectious titer assay

Following infection with Ads, cells were recovered and subjected to 3 cycles of freezing and thawing. After centrifugation, the supernatants were added to HEK293 cells. After incubation for 48 or 72 h, the numbers of cells infected with Ads were analyzed using an Adeno-X Rapid Titer Kit (Clontech).

### Determination of intracellular half-lives of VA-RNAs

The stabilities of VA-RNAs were analyzed using a BRIC kit (MBL, Aichi, Japan) according to the manufacturer’s instructions. Briefly, HeLa-shDicer cells were transfected with pAdVAntage and cultured in doxycycline (Dox)-free or Dox-containing medium (100 ng ml^−1^). After 48 h incubation, the cells were pulse-labeled with 5-bromouridine (BrU) for 24 h, then washed and cultured in fresh medium. At the indicated time points, total RNA including BrU-labeled RNA was extracted from the cells with ISOGEN (Nippon Gene). The BrU-labeled RNA was specifically immunoprecipitated with an antibody against BrU, followed by real-time RT-PCR analysis. The half-lives of VA-RNAI and II were determined from these data.

### Statistical analysis

Statistical significance was determined using Student’s *t*-test. Data are presented as the means ± S.D.

## Results

### Dicer-mediated processing of VA-RNA

**Figure 1:**
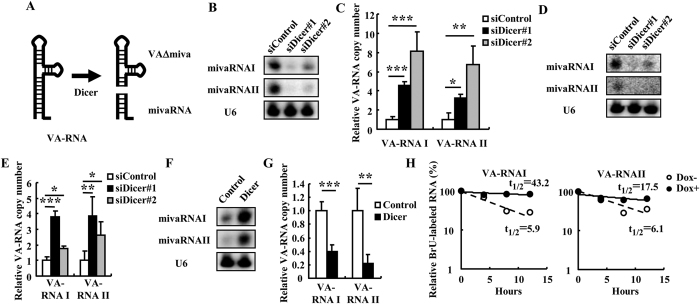
VA-RNA and mivaRNA expression in the Dicer-knockdown cells. (**A**) A schematic diagram of the processing of VA-RNA I by Dicer, produced in VAΔmivaI and mivaRNAI. (**B,C**) HeLa cells were co-transfected with siControl, siDicer#1, or siDicer#2 and a VA-RNA-expressing plasmid (pAdVAntage). The expression levels of mivaRNAs (**B**) and VA-RNAs (**C**) were measured by northern blotting and real-time RT-PCR analysis, respectively, after 48 h incubation. The copy numbers of VA-RNAs in the cells co-transfected with siControl and pAdVAntage were normalized to 1. (**D,E**) HeLa cells were transfected with siControl, siDicer#1, or siDicer#2 and incubated for 48 h, followed by infection with WT-Ad. The expression levels of mivaRNAs (**D**) and VA-RNAs (**E**) were similarly measured. (**F,G**) HeLa cells were co-transfected with a Dicer-expressing plasmid (p3XFLAG-CMV10-Dicer) and a VA-RNA-expressing plasmid (pAdVAntage). The expression levels of mivaRNAs (**F**) and VA-RNAs (**G**) were similarly measured. (**H**) HeLa-shDicer cells were transfected with pAdVAntage and cultured in Dox-free or Dox-containing medium (100 ng ml^−1^). After 48 h incubation, the cells were pulse-labeled with BrU for 24 h, and the copy numbers of BrU-labeled VA-RNAs were measured using a BRIC kit at the indicated time points. t_1/2_: the half-lives of VA-RNA. These data (**C,E,F**) are expressed as the means ± S.D. (n = 4). *p < 0.05, **p < 0.001, ***p < 0.0001 (Student’s *t*-test).

First, in order to examine whether Dicer expression levels were inversely correlated with the copy numbers of VA-RNAs (full-length VA-RNAs), HeLa and H1299 cells were co-transfected with siDicer and VA-RNA-expressing plasmids. Dicer processes full-length VA-RNAs into mivaRNAs and VAΔmiva (Fig. 1A). Dicer mRNA and protein levels were efficiently knocked down following transfection with siDicer#1 and #2 (Supplementary Figure S4A–C). The cell cycle profiles were not largely altered by Dicer knockdown 48 h after transfection (Supplementary Figure S4D), although cell growth was slightly but significantly attenuated by Dicer knockdown (Supplementary Figure S4E). The numbers of dead cells were also not increased by Dicer knockdown (Supplementary Figure S4F). Lower and higher amounts of mivaRNAs (Fig. 1B and Supplementary Figure S5A) and VA-RNAs (Fig. 1C and Supplementary Figure S5B), respectively, were detected in the cells transfected with siDicer#1 and #2 than in the cells transfected with a control siRNA (siControl). Furthermore, when cells were infected with WT-Ad following transfection with siDicer#1 and #2, similar results were found (Fig. 1D,E, and Supplementary Figure S5C). On the other hand, higher and lower amounts of mivaRNAs (Fig. 1F) and VA-RNAs (Fig. 1G), respectively, were detected in the cells transfected with a Dicer-expressing plasmid (p3XFLAG-CMV10-Dicer) than in the cells transfected with a control plasmid. In order to examine the half-lives of VA-RNAs in the cells, HeLa transformants inducibly expressing shDicer (HeLa-shDicer) were transfected with pAdVAntage, a plasmid expressing both VA-RNAI and II. Quantitative RT-PCR analysis and western blotting analysis demonstrated that the knockdown of Dicer in HeLa-shDicer cells was induced at both the mRNA and protein levels by Dox in a dose-dependent manner (Supplementary Figure S6A,B). Dox-induced knockdown of Dicer in HeLa-shDicer cells did not have a significant negative effect on the cell growth (Supplementary Figure S6C). The knockdown of Dicer by Dox treatment in HeLa-shDicer cells significantly prolonged the half-lives of VA-RNAI and II by more than 2-fold (Fig. 1H). Together, these results suggest that the copy numbers of VA-RNAs are negatively regulated by Dicer, which directly processes VA-RNAs into mivaRNAs and VAΔmiva, as previously reported^21^,^30^.

### mivaRNA and VAΔmiva do not support Ad replication

**Figure 2:**
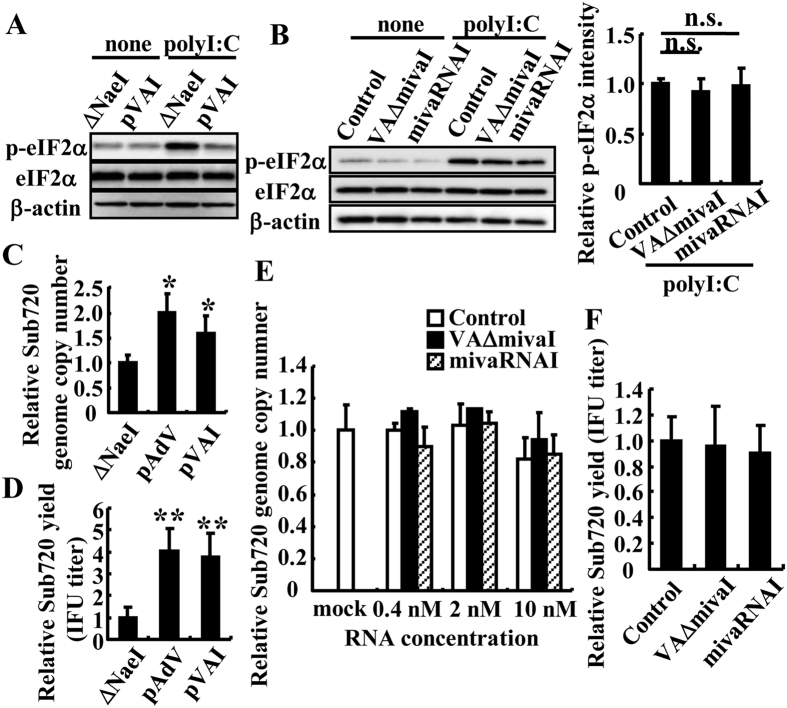
Replication of a VA-RNA–deleted Ad mutant in the cells expressing VA-RNA derivatives. (**A**) HeLa cells were transfected with pAdVAntage-ΔNaeI (ΔNaeI), which is a control plasmid lacking both VA-RNA I and II expression, or pVAI, which is a plasmid expressing only VA-RNA I, for 48 h, followed by transfection with polyI:C (1 μg ml^−1^) for 8 h. Phosphorylated eIF2α levels were determined by western blotting analysis. (**B**) HeLa cells were transfected with *in vitro* transcribed VAΔmivaI or chemically synthesized mivaRNAI for 48 h, followed by transfection with polyI:C (1 μg ml^−1^) for 8 h. Phosphorylated eIF2α levels were evaluated by western blotting analysis. The intensity of p- eIF2α expression in the cells treated with polyI:C was quantified using Image J software. (**C,D**) HeLa cells were transfected with pAdVAntage-ΔNaeI (ΔNaeI), pAdVAntage (pAdV), or pVAI, followed by infection with Sub720 at an MOI of 1. After 24 h incubation, the copy numbers of Sub720 genomic DNA (**C**) and IFU titers of progeny Sub720 (**D**) in the cells were determined by real-time PCR analysis and infectious titer assay, respectively. These data (**C,D**) are expressed as the means ± S.D. (n = 5). (**E,F**) HeLa cells were transfected with VAΔmivaI or mivaRNAI, followed by infection with Sub720 at an MOI of 1. After 24 h incubation, the copy numbers of Sub720 genomic DNA (**E**) and IFU titers of progeny Sub720 (**F**) in the cells were similarly determined. These data (**B,E,F**) are expressed as the means ± S.D. (n = 3–4). *p < 0.01, **p < 0.001 (Student’s *t*-test).

Next, to examine the abilities of the processed forms of VA-RNAs, mivaRNAI and VAΔmivaI, to suppress the phosphorylation of eIF2α, mivaRNAI and VAΔmivaI were each overexpressed in the cells by transfection with chemically synthesized mivaRNAI or *in vitro* transcribed VAΔmivaI, followed by transfection with polyI:C (Fig. 2B). polyI:C is well known to activate PKR, resulting in phosphorylation of eIF2α^31^. Furthermore, to examine the abilities of the processed forms of VA-RNAs to promote Ad replication, these cells were infected with Sub720, an Ad mutant lacking VA-RNA expression (Fig. 2C–F). Northern blotting analysis demonstrated that mivaRNAI expression levels in the cells 24 h after infection with WT-Ad at a multiplicity of infection (MOI) of 5 were comparable to those in cells transfected with chemically synthesized mivaRNAI mimic at a concentration of 2 nM (Supplementary Figure S7A). An *in vitro* reporter gene assay demonstrated that the synthesized mivaRNAI mimic significantly suppressed the target gene expression, similarly to endogenous miRNAs (Supplementary Figure S7B,C). The overexpression of VA-RNA I significantly inhibited the polyI:C-induced phosphorylation of eIF2α (Fig. 2A). On the other hand, eIF2α was efficiently phosphorylated in cells transfected with mivaRNAI or VAΔmivaI (Fig. 2B), suggesting that the production of mivaRNAI or VAΔmivaI by Dicer-mediated processing of VA-RNA I led to a loss of the ability to inhibit eIF2α phosphorylation. Furthermore, the growth of Sub720 was significantly rescued by transfection with the plasmids expressing VA-RNA I (pVAI) or both VA-RNA I and II (pAdVAntage) (Fig. 2C,D). On the other hand, Sub720 replication in cells transfected with mivaRNAI or VAΔmivaI was almost comparable to that in cells transfected with a control mimic (Fig. 2E,F), suggesting that the production of mivaRNAI or VAΔmivaI by Dicer-mediated processing of VA-RNA I was not crucial for Ad replication.

### An Ad expressing mutated mivaRNAI shows normal growth

**Figure 3:**
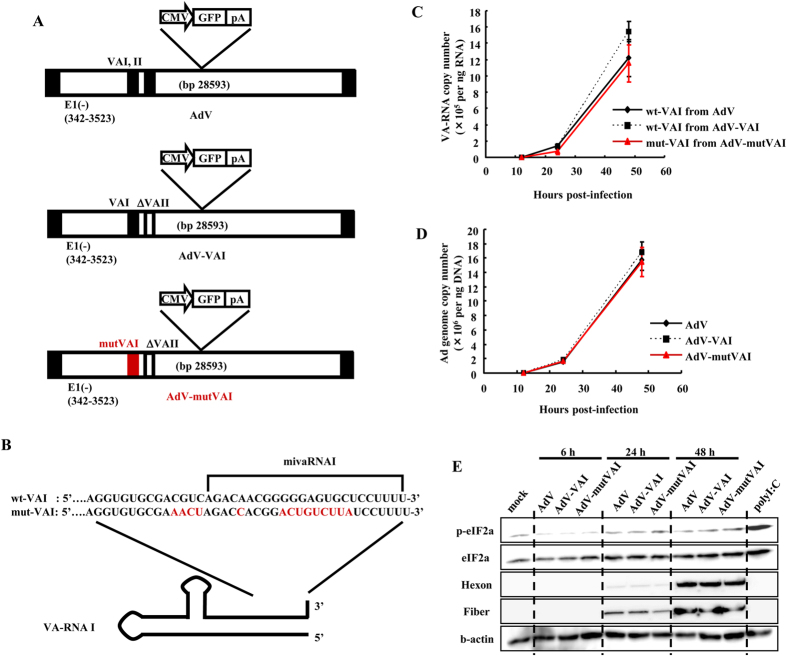
Characterization of an Ad mutant including mutated mivaRNAI sequence. (**A**) A schematic diagram of AdV, AdV-VAI, and AdV-mutVAI. (**B**) A schematic diagram of the 3′-end sequences of wt-VAI and mut-VAI. (**C**) HEK293 cells were transduced with AdV, AdV-VAI, or AdV-mutVAI at an MOI of 1. At the indicated time points, expression levels of VA-RNAs were determined by real-time RT-PCR analysis. (**D**) HEK293 cells were transduced with AdV, AdV-VAI, or AdV-mutVAI at an MOI of 1. At the indicated time points, viral genome copy numbers of AdV, AdV-VAI, or AdV-mutVAI were determined by real-time PCR analysis. These data (**C,D**) are expressed as the means ± S.D. (n = 3). (**E**) Phosphorylated eIF2α protein levels following transduction with AdV, AdV-VAI, or AdV-mutVAI in HEK293 cells. The cells were transduced with AdV, AdV-VAI, or AdV-mutVAI at an MOI of 1 for the indicated hours. HEK293 cells was transfected with polyI:C (1 μg ml^−1^), followed by western blotting analysis after 6 h incubation. Hexon and fiber proteins are major Ad capsid proteins.

In order to examine whether mivaRNAI suppresses the expression of some targeted genes in a sequence-specific manner similarly to an endogenous miRNA for the enhancement of Ad replication, we developed several recombinant Ad mutants possessing mutations in the terminal stem region of the VA-RNA I gene (Fig. 3A). The terminal stem region of the VA-RNA I gene corresponds to the seed sequence of mivaRNAI. AdV-VAI is a VA-RNA II-deleted replication-incompetent Ad vector that expresses VA-RNA I alone. AdV-mutVAI is a mutated Ad vector with mutations in the terminal stem of the VA-RNA I gene in the Ad genome, and it does not express VA-RNA II (Fig. 3B). In silico prediction analysis revealed that the secondary structure of mutated VA-RNA (mut-VAI) was similar to that of wild-type VA-RNA I (wt-VAI) (Supplementary Figure S8A,B). mut-VAI was also processed by Dicer, producing mutated mivaRNAI, which suppressed the expression of the target gene containing the complementary sequences, but not sequences complementary to wt-VAI, in the reporter gene assay (Supplementary Figure S8C). However, the silencing efficiency of AdV-mutVAI-encoded mut-mivaRNAIT in the reporter gene expression (an approximately 40% decrease) was not as high as that of AdV-encoded mivaRNAI (an approximately 60% decrease) (Supplementary Figure S8C). mut-mivaRNAI might be less efficiently incorporated into RISC, compared with mivaRNAI. Real-time RT-PCR analysis demonstrated that copy numbers of wt-VAI and mut-VAI were comparable following transduction with AdV, AdV-VAI, and AdV-mutVAI in HEK293 cells (Fig. 3C). The replication profile of AdV-mutVAI was similar to those of AdV and AdV-VAI (Fig. 3D). Furthermore, phosphorylation of eIF2α was inhibited at similar levels for the all types of Ad vectors tested (Fig. 3E). These results indicate that the mivaRNAI sequence is not crucial for the inhibition of eIF2α phosphorylation or for the support of Ad replication by VA-RNA, even though mivaRNAI might regulate the expression of some unidentified target genes.

### Dicer negatively regulates Ad replication

**Figure 4:**
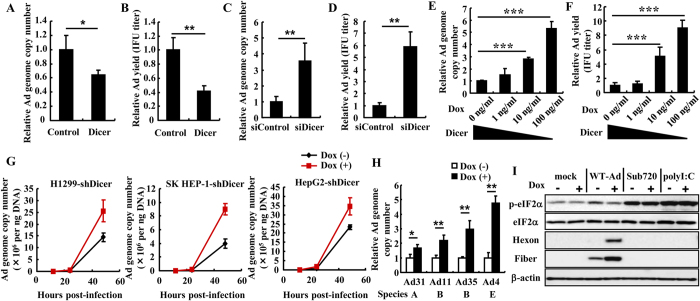
Dicer-mediated suppression of Ad replication. (**A–D**) HeLa cells were transfected with a Dicer-expressing plasmid (p3XFLAG-CMV10-Dicer) (**A,B**) or siRNAs (**C,D**), followed by infection with WT-Ad at an MOI of 5. After 24 h incubation, the copy numbers of WT-Ad genomic DNA (**A,C**) and the IFU titers of progeny WT-Ad (**B,D**) in the cells were determined by real-time PCR analysis and infectious titer assay, respectively. (**E,F**) HeLa-shDicer cells were cultured in Dox-free or Dox-containing medium at the indicated concentrations for 48 h, followed by infection with WT-Ad at an MOI of 5. After 24 h incubation, the copy numbers of WT-Ad genomic DNA (**E**) and IFU titers of progeny WT-Ad (**F**) in the cells were similarly determined. (**G**) H1299-shDicer, SK HEP-1-shDicer, and HepG2-shDicer cells were cultured in Dox-free or Dox-containing (100 ng ml^−1^) medium for 48 h, followed by infection with WT-Ad at an MOI of 5. At the indicated time point, copy numbers of WT-Ad genomic DNA were determined by real-time PCR analysis. (**H**) HeLa-shDicer cells were cultured in Dox-free or Dox-containing (100 ng ml^−1^) medium for 48 h, followed by infection with Ad31, Ad11, Ad35, or Ad4 at 100 virus particles (VP) per cell. After 24 h incubation, the copy numbers of each Ad genomic DNA were determined by real-time PCR analysis. These data (**A–H**) are expressed as the means ± S.D. (n = 3–4). (**I**) Phosphorylated eIF2α protein levels following infection with WT-Ad or Sub720 in HeLa-shDicer cells were analyzed by western blotting analysis. The cells were cultured in Dox-free or Dox-containing (100 ng ml^−1^) medium for 48 h, followed by infection with WT-Ad or Sub720 at an MOI of 5 for 24 h. HeLa-shDicer cells cultured in Dox-free or Dox-containing (100 ng ml^−1^) medium for 48 h were transfected with polyI:C (1 μg ml^−1^), followed by western blotting analysis after 6 h incubation. Hexon and fiber proteins are major Ad capsid proteins. *p < 0.05, **p < 0.01, ***p < 0.001 (Student’s *t*-test).

As shown in Fig. 1, higher amounts of VA-RNAs derived from WT-Ad were found in Dicer-knockdown cells than in control cells. In order to examine whether the promotion or suppression of Dicer-mediated VA-RNA processing altered Ad replication, Dicer-overexpressing and -knockdown cells were infected with WT-Ad. Transfection with a Dicer-expressing plasmid resulted in more than 300-fold and 30-fold higher amounts of Dicer mRNA in HeLa and H1299 cells, respectively (Supplementary Figure S9A). Copy numbers of the WT-Ad genome were reduced by about 35% and 60% in Dicer-overexpressing HeLa and H1299 cells, respectively, compared with cells transfected with a control plasmid (Fig. 4A and Supplementary Figure S9B). IFU titers of progeny WT-Ad were also reduced by more than 50% in Dicer-overexpressing HeLa cells (Fig. 4B). In contrast, approximately 3.5- and 2.3-fold higher copy numbers of the WT-Ad genome were found in HeLa and H1299 cells, respectively, when Dicer was knocked down (Fig. 4C and Supplementary Figure S9C). IFU titers of progeny WT-Ad were also increased by approximately 6-fold in Dicer-knockdown HeLa cells (Fig. 4D). As shown in Supplementary Figure S4E, cell growth was slightly attenuated by transfection with siDicer#1; however, the promotion of Ad replication by knockdown of Dicer was not attributed to the slight attenuation of cell growth itself, because Ad replication levels were not altered in the cells that showed low growth rates due to the low FBS concentration (Supplementary Figure S9D,E). In order to further demonstrate that the knockdown of Dicer enhances Ad replication, HeLa-shDicer cells were infected with WT-Ad. When HeLa-shDicer cells were infected with WT-Ad in the presence of various concentrations of Dox, copy numbers of the WT-Ad genome and IFU titers of progeny WT-Ad in the cells were inversely correlated with the Dicer expression levels (Fig. 4E,F). WT-Ad also replicated more efficiently in the other human cell lines that inducibly expressed shDicer in the presence of Dox (100 ng ml^−1^) than in the absence of Dox (Fig. 4G). Upregulation of Ad replication in Dicer-knockdown cells was also found for the other human Ad serotypes (Ad serotype 31 (Ad31, species A), serotype 11 (Ad11, species B), serotype 35 (Ad35, species B), and serotype 4 (Ad4, species E)) (Fig. 4H). In order to rule out the possibility that elevation of Ad replication by Dicer knockdown was caused by off-target effects, a silent mutation was introduced in the shDicer-targeted sequence of the Dicer gene (mDicer) in the plasmid to make the Dicer mRNA resistant to shDicer. Overexpression of mDicer cancelled the shDicer-mediated elevation in the copy numbers of VA-RNAI and the Ad genome in WT-Ad-infected HeLa-shDicer cells in the presence of Dox (100 ng ml^−1^) (Supplementary Figure S10). These results indicate that Dicer expression negatively regulates Ad replication.

In order to examine the Ad replication profile in a tumor cell subline showing lower Dicer expression than a parent tumor cell line, MCF-7 cells resistant to adriamycin (MCF-7/ADR cells) were infected with WT-Ad. Previous studies reported that malignancies in tumor tissues were inversely correlated with Dicer expression levels in tumor tissues^32^. Five-fold lower levels of Dicer mRNA were detected in MCF-7/ADR cells than in parent MCF-7 cells (Supplementary Figure S11A). There were approximately 14-fold higher copy numbers of the Ad genome in MCF-7/ADR cells than in parent MCF-7 cells (Supplementary Figure S11B). These results indicate that Ad also replicates more efficiently in tumor cell sublines expressing low levels of Dicer.

VA-RNAs block PKR activation, leading to inhibition of phosphorylation of eIF2α and efficient replication of Ad^8^,^15^. In order to examine whether an increase in the copy numbers of VA-RNA by Dicer knockdown leads to a reduction in Ad-induced phosphorylation of eIF2α, HeLa-shDicer cells were cultured in the presence or absence of Dox (100 ng ml^−1^), followed by infection with WT-Ad or Sub720. In the absence of Dox, Sub720 significantly induced eIF2α phosphorylation. Phosphorylated eIF2α (p-eIF2α) levels in the cells infected with WT-Ad were lower than those in the cells infected with Sub720 (Fig. 4I), probably because Sub720 did not inhibit the Ad-induced activation of PKR due to the lack of VA-RNA expression. Interestingly, p-eIF2α levels were significantly lower in WT-Ad-infected HeLa-shDicer cells in the presence of Dox than in the absence of Dox, resulting in the efficient production of Ad proteins, including hexon and fiber proteins, in the presence of Dox. Dicer knockdown alone did not alter the amounts of eIF2α or p-eIF2α in mock-transfected cells or polyI:C-transfected cells. Copy numbers of the WT-Ad genome were increased by approximately 3.5-fold in HeLa cells transfected with siPKR, compared with cells transfected with the siControl, probably due to blockade of PKR-mediated phosphorylation of eIF2α (Supplementary Figure S12A,B). In addition, PKR knockdown significantly restored the Ad replication in Dicer-overexpressing cells (Supplementary Figure S12C). These results indicate that in Ad-infected cells, Dicer-mediated processing of VA-RNA causes a reduction in the amounts of VA-RNA, leading to efficient phosphorylation of eIF2α and the inhibition of Ad replication. In contrast, Dicer knockdown resulted in increased amounts of VA-RNA, leading to the inhibition of eIF2α phosphorylation and the promotion of Ad replication.

### Involvement of Ago2 in Ad replication

After Dicer-mediated processing in post-transcriptional silencing, miRNA is incorporated into RISC, which is mainly composed of Ago2, leading to inhibition of the expression of target genes^1^,^2^. Dicer knockdown resulted in efficient replication of Ad as shown above; however, Dicer knockdown could have enhanced Ad replication by perturbing miRNA-mediated post-transcriptional silencing. In order to examine this possibility, Ago2-knockdown cells were infected with WT-Ad. Ago2 was significantly knocked down at both the mRNA and protein levels following transfection with siAgo2 (Supplementary Figure S13A,B). Although siAgo2 did not alter the copy numbers of VA-RNAs in HeLa cells co-transfected with pAdVAntage (Supplementary Figure S13C) and in those infected with WT-Ad (Supplementary Figure S13D), the copy numbers of mivaRNAI were significantly reduced by Ago2 knockdown (Supplementary Figure S13E). Conversely, over-expression of Ago2 resulted in an apparent elevation of mivaRNAI copy numbers (Supplementary Figure S13E). Approximately 2-fold higher levels of both the WT-Ad genome copy numbers and IFU titers of progeny WT-Ad were found in HeLa cells when Ago2 was knocked down (Supplementary Figure S13F,G); however, the levels of enhancement of Ad replication by Ago2 knockdown (about two-fold) were lower than those by Dicer knockdown (about four-fold). In order to further examine whether perturbation in miRNA-mediated post-transcriptional silencing *via* Dicer knockdown was involved in the promotion of Ad replication in Dicer-knockdown cells, WT-Ad was added to the Ago2/Dicer double-knockdown cells. Both Dicer and Ago2 were significantly knocked down at the mRNA levels following treatment with Dox and transfection with siAgo2 (Supplementary Figure S13H). Transfection with siAgo2 alone significantly altered miRNA expression profiles compared with the siControl treatment (Supplementary Figure S13I). On the other hand, the miRNA expression profiles were not significantly altered in Ago2/Dicer double-knockdown cells compared with Ago2-knockdown cells (Supplementary Figure S13J and Table S1), although the expression profiles of miRNAs at low expression levels were slightly altered. A reporter assay demonstrated that post-transcriptional gene silencing of the representative miRNAs (miR-27a and let-7a) was cancelled by transfection with siAgo2 alone, but Dicer knockdown did not further restore reporter gene expression (Supplementary Figure S13K). These results indicate that the knockdown of Ago2 alone sufficiently inhibited miRNA-mediated post-transcriptional gene silencing under these experimental conditions and that the knockdown of Dicer, in addition to that of Ago2, had no significant effect on the miRNA expression profiles. However, the double knockdown of both Ago2 and Dicer promoted Ad replication approximately 10-fold more efficiently than the knockdown of Ago2 alone (Supplementary Figure S13L). These results indicate that the knockdown of Dicer significantly enhances Ad replication regardless of global changes in miRNA expression profiles.

## Discussion

It has been unclear whether RNAi functions as an antivirus system in mammalian cells, especially for DNA viruses. Rather, DNA viruses, including herpesviridae, have been demonstrated to express virus-derived miRNAs and to utilize post-transcriptional gene silencing for their survival^1^,^2^,^5^,^6^. The aim of this study is to examine whether Dicer acts as an antivirus system for Ad in mammalian cells through Dicer-mediated processing of VA-RNA. Previous studies have reported that VA-RNAs were processed by Dicer, leading to the production of mivaRNAs^17^,^18^,^19^,^27^,^33^; however, those studies did not clarify whether this processing has positive or negative effects on Ad replication. In this study, we have demonstrated that in Dicer-knockdown cells, significantly higher amounts of full-length VA-RNAs were found (Fig. 1), resulting in the inhibition of eIF2α phosphorylation (Fig. 4I), which was associated with the inhibition of the translation of viral mRNA and/or with the inhibition of cell apoptosis^7^,^34^,^35^,^36^,^37^. Dicer-mediated processed forms of VA-RNA I, VAΔmivaI and mivaRNAI, did not apparently enhance Ad replication (Figs 2 and 3). These results suggest that Dicer functions as an antiviral system against Ad *via* the processing of VA-RNAs.

Viral dsRNAs derived from RNA viruses are cleaved by Dicer, resulting in virus-derived siRNAs in plants, nematodes, and insect cells^1^,^2^. On the other hand, Dicer in mammalian somatic cells would less effectively process viral long dsRNA, which is considered to be produced during replication of the RNA virus genome, into siRNA, compared with Dicer in plants, nematodes, and insect cells, although efficient Dicer-mediated processing of virus-derived long dsRNA has been observed in mouse embryonic stem cells and BHK-21 cells^3^,^4^. The N-terminal DExD helicase domain in the Dicer isoform which is expressed in mammalian somatic cells inhibits the efficient processing of long dsRNA into siRNA^38^,^39^, while the Dicer isoform expressed in oocytes is lacking the N-terminal DExD helicase domain, leading to efficient production of siRNA from long dsRNA^40^. Although there have been several reports demonstrating that replication of several RNA viruses is elevated by Dicer knockdown in mammalian cells^41^,^42^, Dicer might inefficiently cleave virus-derived dsRNA or, alternatively, knockdown of Dicer might enhance replication of the RNA viruses *via* an unknown mechanism other than the cleavage of virus-derived dsRNA. On the other hand, the mammalian Dicer isoform mediates efficient processing of pre-miRNA into miRNA^38^. VA-RNAs are efficiently processed by Dicer in mammalian cells due to their pre-miRNA-like secondary structure (Supplementary Figure S8A), leading to inhibition of Ad replication. Other virus-derived RNAs with pre-miRNA-like secondary structures would be efficiently processed by Dicer in mammalian cells. For example, Epstein-Barr (EB) virus expresses EBERs, which are pre-miRNA-like non-coding RNAs. EBERs are processed by Dicer into EBER-derived miRNAs similarly to VA-RNAs^43^.

Dicer-mediated processing of VA-RNA I produces mivaRNAI and a truncated form of VA-RNA I, VAΔmivaI. As shown in Fig. 2, neither VAΔmivaI nor mivaRNAI induced the phosphorylation of eIF2α following transfection, suggesting that PKR was not activated by the cleaved VA-RNAs in cultured cells. In addition, VAΔmivaI neither suppresses the phosphorylation of eIF2α nor supports Ad replication, suggesting that VAΔmivaI did not have the ability to inhibit PKR activation. These results contradict those of a previous report. Using an *in vitro* PKR autophosphorylation inhibition assay, Wahid *et al.* demonstrated that VAΔmivaI had the ability to inhibit PKR activation as well as the full-length VA-RNA I; however, that study did not examine the ability of VAΔmivaI to inhibit PKR activation in the culture cells^30^. The terminal-stem region, which is converted to mivaRNAs by Dicer-mediated processing, in VA-RNA I plays an important role in the stability of VA-RNA I^15^,^30^. Destabilization of the secondary structure of VAΔmivaI would be induced by Dicer-mediated processing in the cells, leading to the degradation of VAΔmivaI and the loss of inhibition of PKR phosphorylation. Actually, we attempted to detect VAΔmivaI in the cells transfected with a VA-RNA-expressing plasmid by northern blotting analysis; however VAΔmivaI could not be detected, probably due to a rapid degradation, despite the efficient detection of VA-RNAs and mivaRNAs.

Various types of viruses possess anti-RNAi systems. For example, HIV-1 encodes the Tat protein, which inhibits the function of Dicer-mediated cleavage of dsRNA into siRNA^44^. VA-RNAs have been also demonstrated to function as anti-RNAi systems. Benkirane *et al.* demonstrated that VA-RNA inhibited the export of Dicer mRNA from the nucleus to the cytoplasm by competitive binding for Exportin5 between Dicer mRNA and VA-RNA, leading to the reduction of Dicer protein levels after Ad infection^45^. This function of Dicer is highly crucial for Ad infection, because Ad replication is promoted by the inhibition of Dicer, as shown in the present study. The previous reports demonstrated that VA-RNAs promoted Ad growth by interaction with PKR as a main target of VA-RNAs^7^,^8^,^12^,^13^. These results suggest that VA-RNA-mediated inhibition of Dicer expression leads to increased VA-RNA copy numbers in Ad-infected cells, resulting in more efficient inhibition of PKR by VA-RNAs and to the enhancement of Ad replication.

In the present experiments, knockdown of Dicer did not largely alter the cell cycle profiles and growth of the cultured cell lines (Supplementary Figures S4D,E and 6C); however, Dicer knockdown should have certain effects on cellular homeostasis *via* its perturbation of miRNA expression profiles. In addition to effects on cellular homeostasis, the alteration of miRNA expression profiles by Dicer knockdown would lead to promotion of virus replication *via* various mechanisms. Ostermann *et al.* demonstrated that knockdown of Dicer resulted in alteration of miRNA expression profiles, leading to the impairment of upregulation of interferon (IFN)-stimulated genes and the enhancement of mouse cytomegalovirus (CMV) replication following infection^46^. Otsuka *et al.* reported that expression of miR-24 and miR-93, which directly target the genome of vesicular stomatitis virus (VSV) *via* post-transcriptional gene silencing, was reduced in Dicer-deficient mice, leading to increased replication of VSV^47^. Ad replication would be regulated by endogenous miRNAs. As shown in Supplementary Figure S13F,G, knockdown of Ago2, which is crucial for miRNA-mediated post-transcriptional silencing, led to the promotion of Ad replication, suggesting that inhibition of miRNA-mediated post-transcriptional silencing by Ago2 knockdown promotes Ad replication *via* unknown mechanism. Knockdown of Ago2 would lead to the restoration of several gene expressions that promote Ad replication, leading to up-regulation of Ad replication. Although further studies are required for the elucidation of endogenous miRNA-mediated regulation of Ad replication, the effects of alteration of miRNA expression profiles *via* Dicer knockdown on Ad replication would be smaller than those of other factors, including the inhibition of VA-RNA processing. The reporter assay revealed that the knockdown of both Ago2 and Dicer did not further restore reporter gene expression compared with the knockdown of Ago2 alone, indicating that the latter sufficiently cancelled the miRNA-mediated post-transcriptional gene silencing (Supplementary Figure S13K). In addition, there were no significant differences in the expression profiles of miRNAs, especially of highly expressed miRNAs, between cells showing knockdown of Ago2 alone and those showing knockdown of both Ago2 and Dicer (Supplementary Figure S13I,J). Despite of these findings, the knockdown of both Ago2 and Dicer more efficiently promoted Ad replication than did the knockdown of Ago2 alone (Supplemental Figure S13L), suggesting that the inhibition of miRNA-mediated post-transcriptional gene silencing *via* Dicer knockdown did not contribute substantially to the enhancement of Ad replication in Dicer-knockdown cells.

Transfection with chemically synthesized mivaRNAI suppressed the expression of reporter gene containing mivaRNAI-targeted sequences in the 3′-UTR (Supplementary Figure S7C). Similar results were obtained following transduction with a replication-incompetent Ad vector (Supplementary Figure S8C). These results indicate that mivaRNAI is incorporated into RISC and functions as an miRNA. However, chemically synthesized mivaRNAI did not promote or suppress Ad replication, indicating that mivaRNAI is dispensable for Ad replication, although a recent study reported several target genes of mivaRNAI^48^. In addition, these results indicated that RISC containing mivaRNAI did not knock down VA-RNA I through RNAi. This is in contrast to antiviral RNAi against RNA viruses, in which Dicer processes the virus-derived dsRNA genome, producing siRNA that guides RISC to degrade complementary viral RNA.

Ago2 knockdown significantly reduced the copy numbers of mivaRNAI (Supplementary Figure S13E), although the copy numbers of full-length VA-RNAI were not increased by Ago2 knockdown (Supplementary Figure S13C,D). On the other hand, overexpression of Ago2 significantly increased the copy numbers of mivaRNAI (Supplementary Figure S13E). Argonaute proteins are known to be highly crucial for intracellular stability of miRNAs^49^. Copy numbers per cell of argonaute proteins, including Ago1 and Ago2, have been reported to be approximately 10^5^ molecules per cell^50^, which is much lower than the VA-RNA copy numbers per cell (>10^8^ molecules per cell). These findings suggest that VA-RNAs are more efficiently cleaved by Dicer, leading to the production of large numbers of mivaRNAs; however, not all mivaRNAs are incorporated into RISC. mivaRNAs which are not incorporated into RISC are susceptible to degradation.

We have demonstrated here the antiviral function of Dicer in Ad infection in the cultured cells; however, it has remained to be clarified whether Dicer shows the antiviral function *in vivo*. We did not use a mouse model in this study, since human Ads cannot replicate in mice^51^. In addition, Dicer knockout mice are embryonic lethal^52^. A Syrian hamster would be a promising model to examine the *in vivo* antiviral function of Dicer in Ad infection because human Ads can replicate in a Syrian hamster^53^.

In summary, we have demonstrated that Dicer-mediated processing of VA-RNAs results in the loss of VA-RNA-mediated PKR inhibition activity and the subsequent reduction of Ad replication, indicating that Dicer functions as an antiviral system even against DNA viruses in mammalian cells.

## Additional Information

**How to cite this article**: Machitani, M. *et al.* Dicer functions as an antiviral system against human adenoviruses *via* cleavage of adenovirus-encoded noncoding RNA. *Sci. Rep.* **6**, 27598; doi: 10.1038/srep27598 (2016).

## Supplementary Material

Supplementary Information
