# Supplementary material for: Dicer functions as an antiviral system against human adenoviruses via cleavage of adenovirus-encoded noncoding RNA
Source: Sci Rep. 2016 Jun 7;6:27598. doi: 10.1038/srep27598 (PMC4895142; doi:10.1038/srep27598)
Supplement: Supplementary Information [file srep27598-s1.pdf]

SUPPLEMENTARY INFORMATION

Dicer functions as an antiviral system against human adenoviruses *via* cleavage of adenovirus-encoded noncoding RNA

Mitsuhiro Machitani, Fuminori Sakurai, Keisaku Wakabayashi, Kyoko Tomita, Masashi Tachibana, Hiroyuki Mizuguchi

SUPPLEMENTARY MATERIALS and METHODS

Plasmids

A self-inactivating lentivirus vector (SIN-LV) construct (CS-CDF-H1tetO-shDicer-ETR), which inducibly expresses shDicer and constitutively expresses tetracycline repressor and monomeric red fluorescent protein (mRFP1) as a single transcript linked with a 2A-self-cleaving peptide (Supplementary Figure S2), was constructed as follows. pENTR4-H1TetOx1 (1) was designed to express shRNA by the insertion of oligonucleotides encoding shRNA into the *Bgl*II/*Xba*I site. In order to suppress leaky expression without tetracycline, one more tetracycline-operator (tetO) sequence was inserted into the H1tetO promoter, resulting in pENTR-H1T2. A *Bgl*II/*Xba*I fragment of pENTR-H1T2 was ligated with oligonucleotides shDicer-S and shDicer-AS, resulting in pENTR-H1T2-shDicer. The cassette containing the H1T2 promoter plus the shDicer was incorporated into a self-inactivating lentivirus vector (SIN-LV) construct (CS-RfA-ETR) (1), resulting in CS-H1T2-shDicer-ETR. The sequences of shDicer-S and shDicer-AS are shown in Supplementary Table S2.

Reporter plasmids, psiCHECK-2-miR-27aT and -let-7aT, containing two copies of a sequence complementary to miR-27a and let-7a, respectively, in the 3'-UTR of the RLuc gene, were constructed as follows. An *Xho*I/*Not*I fragment of psiCHECK-2 (Promega) was ligated with oligonucleotides encoding sequences complementary to miR-27a, miR-27aT-S and miR-27aT-AS, resulting in psiCHECK-2-miR-27aT. psiCHECK-2-let-7aT was similarly constructed using the corresponding oligonucleotides. The sequences of the oligonucleotides are shown in Supplementary Table S2.

p3XFLAG-CMV10-mDicer, a plasmid expressing a silently-mutated Dicer (mDicer) which is resistant to shDicer, was constructed as follows: the shDicer-targeted sequence in p3XFLAG-CMV10-Dicer was mutated by using a QuikChange Site-Directed Mutagenesis Kit (Agilent Technologies) and the primers mDicer-F and mDicer-R, resulting in p3XFLAG-CMV10-mDicer.

Preparation of SIN-LV

A lentivirus vector (LV) pseudotyped with vesicular stomatitis virus G glycoprotein was prepared according to a previously described method with some modification (2,3). Briefly, 293T cells were transfected with three plasmids: the packaging construct (pCAG-HIVgp), VSV-G and Rev-expressing construct (pCMV-VSV-G-RSV-Rev), and SIN vector construct (CS-H1T2-shDicer-ETR). The vector supernatant was concentrated by ultracentrifugation, and the pellet was resuspended in Hanks' balanced salt solution. Vector titers, which can be detected by enhanced mRFP1 expression, were

determined by infection of HeLa cells with serial dilutions of the vector stocks, followed by fluorescence-activated cell sorter (FACS) analysis for mRFP1-positive cells. pCAG-HIVgp and pCMV-VSV-G-RSV-Rev are also available from the RIKEN BioResource Center.

Cell viability assay

After treatment with siRNAs or Dox, cell viabilities were determined at the indicated time points by staining with AlamarBlue (Invitrogen) according to the manufacturer's instructions.

Secondary structure prediction of VA-RNA

We have used mfold for calculating nucleic acid secondary structures by free energy minimization (4). RNA folding was performed at the RNA Mfold server (http://mfold.rna.albany.edu/?q=mfold/rna-folding-form).

Analysis of cell cycle profile

Following transfection with siControl or siDicer, cells were fixed with 66% ethanol and stained with propidium iodide (PI)/RNase staining buffer (BD Pharmingen, San Diego, CA). The stained cells were analyzed by flow cytometry.

miRNA microarray analysis

miRNA microarray analysis was performed by Filgen Co (Aichi, Japan). Briefly, a total RNA samples were biotinylated using FlashTag Biotin HSR RNA labeling Kit (Affymetrix, Santa Clara, CA) according the user manual. The biotinylated samples were hybridized for 18 h on GeneChip miRNA 4.0 Array (Affymetrix). GeneChips were washed and stained in Fluidics Station 450 (Affymetrix). GeneChips were scanned using GeneChip Scanner 3000 7G (Affymetrix). The data were normalized and analyzed using Affymetrix Expression Console Software. The data of miRNA expression profiles in HeLa-shDicer cells showing knockdown of both Ago2 and Dicer have been submitted to the gene expression omnibus (GEO). The GEO accession number is GSE67992.

Reference

1. Machitani, M., Katayama, K., Sakurai, F., Matsui, H., Yamaguchi, T., Suzuki, T., Miyoshi, H., Kawabata, K. and Mizuguchi, H. (2011) Development of an adenovirus vector lacking the expression of virus-associated RNAs. *J Control Release*, **154**, 285-289.
2. Katayama, K., Wada, K., Miyoshi, H., Ohashi, K., Tachibana, M., Furuki, R., Mizuguchi, H., Hayakawa, T., Nakajima, A., Kadowaki, T. *et al.* (2004) RNA interfering approach for clarifying the PPARgamma pathway using lentiviral vector expressing short hairpin RNA. *FEBS Lett*, **560**, 178-182.
3. Miyoshi, H., Smith, K.A., Mosier, D.E., Verma, I.M. and Torbett, B.E. (1999) Transduction of human CD34+ cells that mediate long-term engraftment of NOD/SCID mice by HIV vectors. *Science*, **283**, 682-686.
4. Zuker, M. (2003) Mfold web server for nucleic acid folding and hybridization prediction. *Nucleic Acids Res*, **31**, 3406-3415.

SUPPLEMENTARY FIGURES

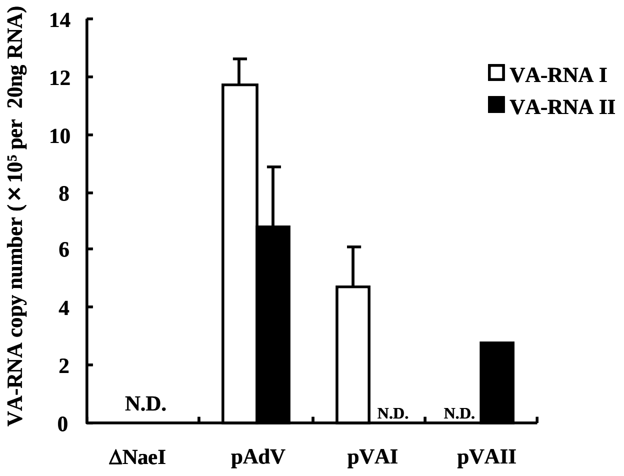

Figure S1

VA-RNA expression following transfection with pAdVAntage-ΔNaeI, pAdVAntage, pVAI, and pVAII.

HeLa cells were transfected with pAdVAntage-ΔNaeI (ΔNaeI), pAdVAntage (pAdV), pVAI, and pVAII. After 48 h incubation, expression levels of VA-RNAs were determined by real-time RT-PCR analysis. The data are expressed as the means ± S.D. (n=3).

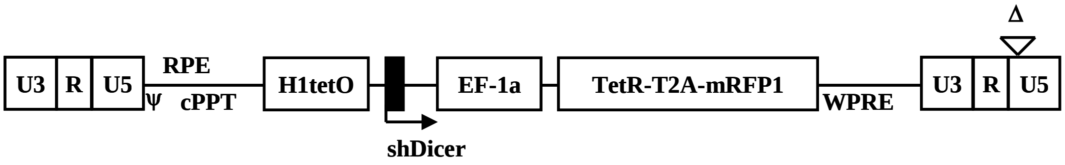

Figure S2

The structure of a lentivirus vector carrying a cassette inducibly expressing shDicer under the control of a tetracycline-dependent H1 promoter (LV-H1tetO-shDicer-ETR).

The SIN-LV was prepared according to the previously described method with some modifications; refer to *Supplementary Methods*. Ψ: packaging signal; RRE: rev responsive element; cPPT: central polypurine tract; EF-1α: human elongation factor 1α promoter; TetR: tetracycline repressor; T2A: self-cleaving 2A peptides; mRFP1: monomeric red fluorescent protein; WPRE: woodchuck hepatitis virus post-transcriptional regulatory element; Δ: deletion of 133 bp in the U3 region of the 3' long terminal repeat.

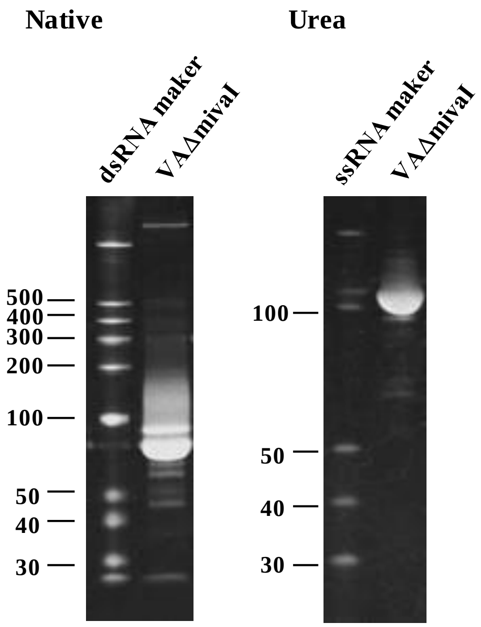

Figure S3

Gel electrophoresis of VAΔmivaI synthesized by *in vitro* transcription.

VAΔmivaI synthesized by *in vitro* transcription was electrophoresed on polyacrylamide and TBE-Urea gels under nondenaturing and denaturing conditions, respectively, followed by ethidium bromide staining.

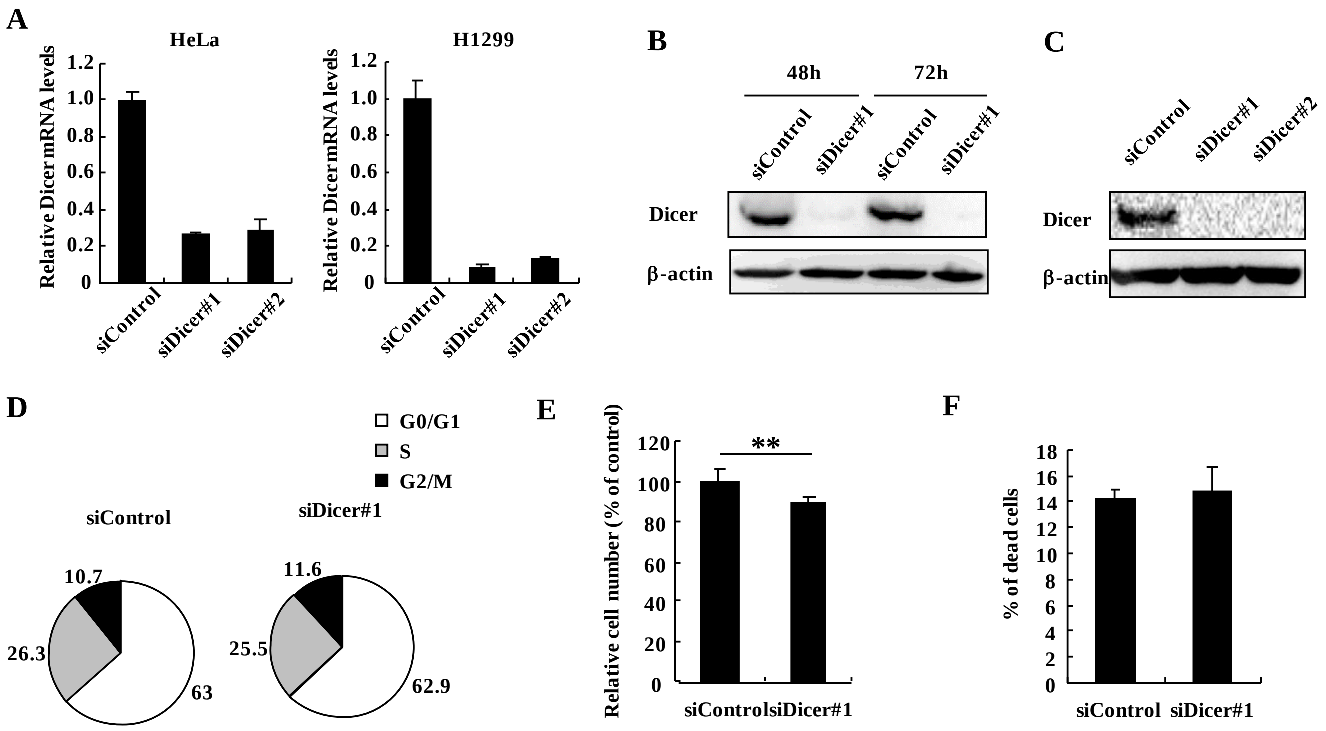

Figure S4

VA-RNA and mivaRNA expression in the Dicer-knockdown cells.

(A) HeLa and H1299 cells were transfected with siControl, siDicer#1, or siDicer#2. After 48 h incubation, Dicer mRNA levels were determined by real-time RT-PCR analysis. Dicer mRNA levels in the cells transfected with siControl were normalized to 1. (B) HeLa cells were transfected with siControl or siDicer#1. After 48 or 72 h incubation, the Dicer protein levels were determined by western blotting analysis. (C) HeLa cells were transfected with siControl, siDicer#1, or siDicer#2. After 48 h incubation, the Dicer protein levels were determined by western blotting analysis. (D) HeLa cells were transfected with siControl or siDicer#1. After 48 h incubation, the cell cycle profiles were analyzed by flow cytometry. (E) HeLa cells were transfected with siControl or siDicer#1. After 48 h incubation, the cell numbers were determined by Alamar blue assay. The data on cells transfected with siControl were normalized to 100%. (F) HeLa cells were transfected with siControl or siDicer#1. After 48 h incubation, the percentage of dead cells was determined under a microscope following trypan blue staining. These data (A, E, F) are expressed as the means ± S.D. (n=3-4). **p<0.001.

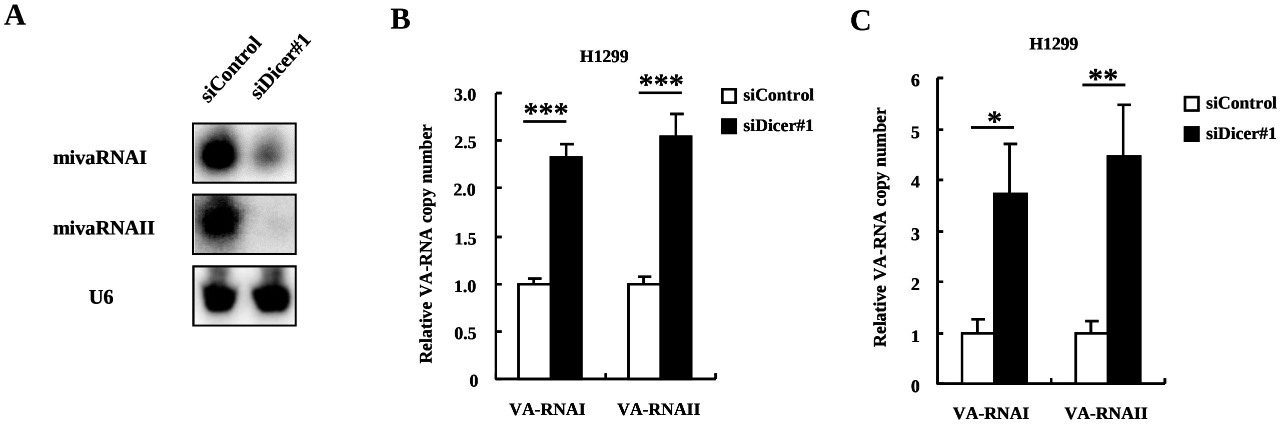

Figure S5

VA-RNA and mivaRNA expression in the Dicer-knockdown H1299 cells.

(A, B) H1299 cells were co-transfected with siControl or siDicer#1 and a VA-RNA-expressing plasmid (pAdVAntage). The expression levels of mivaRNAs (A) and VA-RNAs (B) were measured by northern blotting and real-time RT-PCR analysis, respectively, after 48 h incubation. The copy numbers of VA-RNAs in the cells co-transfected with siControl and pAdVAntage were normalized to 1. (C) H1299 cells were transfected with siControl or siDicer#1 and incubated for 48 h, followed by infection with WT-Ad. The copy numbers of VA-RNAs were measured by real-time RT-PCR analysis 24 h after infection with WT-Ad. These data are expressed as the means ± S.D. (n=4). *p<0.01, **p<0.001, ***p<0.0001.

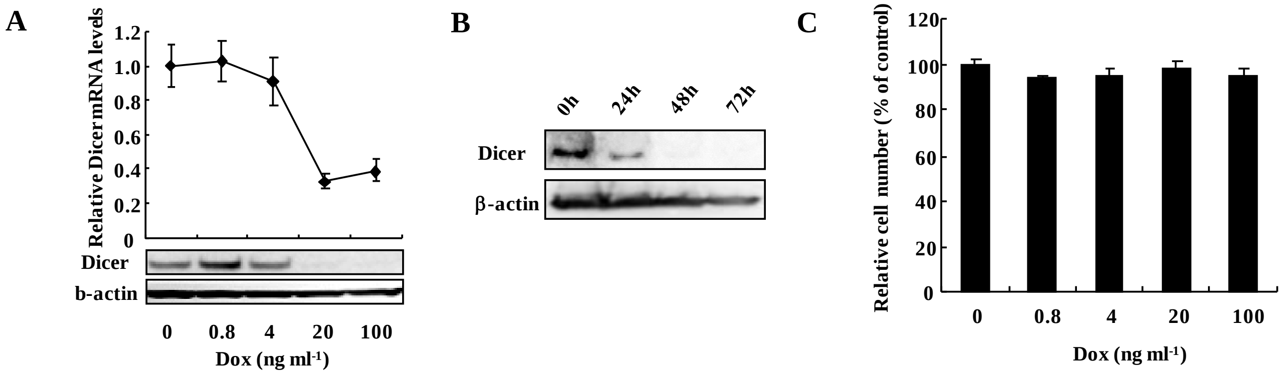

Figure S6

Dox-induced knockdown of Dicer in HeLa-shDicer cells.

(A) HeLa-shDicer cells were cultured in Dox-free or Dox-containing medium at the indicated concentrations. After 48 h incubation, the Dicer mRNA and protein levels were determined by real-time RT-PCR analysis and western blotting analysis, respectively. (B) HeLa-shDicer cells were cultured in Dox-containing medium (100 ng ml^{-1}). At the indicated time points, Dicer protein levels were determined by western blotting analysis. (C) HeLa-shDicer cells were cultured in Dox-free or Dox-containing medium at the indicated concentrations. After 48 h incubation, the cell numbers were determined by Alamar blue assay. The data on cells cultured in Dox-free medium were normalized to 100%. The data are expressed as the means ± S.D. (n=4).

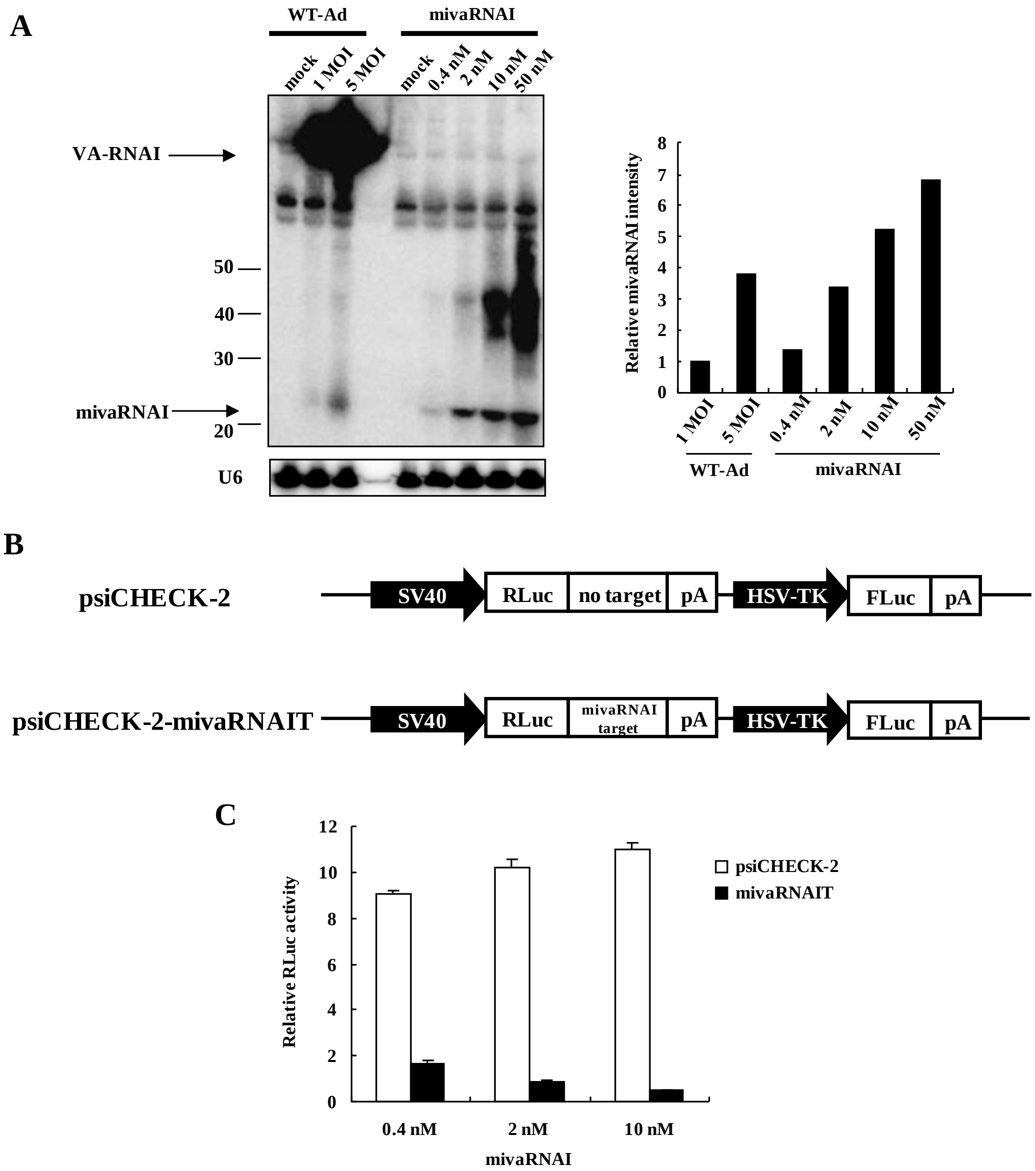

Figure S7

Suppression of the target gene expression by chemically synthesized mivaRNAI.

(A) HeLa cells were infected with WT-Ad at the indicated MOI or transfected with chemically synthesized mivaRNAI at the indicated RNA concentration. After 24 h incubation, total RNA was isolated from the cells, followed by northern blotting analysis. The intensity of mivaRNAI expression in cells treated with WT-Ad or chemically synthesized mivaRNAI was quantified using Image J software. (B) A schematic diagram of reporter plasmids, psiCHECK-2 and psiCHECK-2-mivaRNAIT. psiCHECK-2-mivaRNAT has two copies of a sequence perfectly complementary to mivaRNAI in the 3'-UTR of the renilla luciferase (RLuc) gene. RLuc activity was normalized by firefly luciferase (FLuc) activity. SV40: simian virus 40 early enhancer/promoter; HSV-TK: herpes simplex virus-thymidine kinase promoter; pA: poly A signal. (C) Chemically synthesized mivaRNAI was co-transfected with a control reporter plasmid (psiCHECK-2) or psiCHECK-2-mivaRNAT (mivaRNAIT) into HeLa cells. After 24 h incubation, luciferase activity was determined. The data show RLuc activity normalized by FLuc activity. The data are expressed as the means ± S.D. (n=4).

A B C

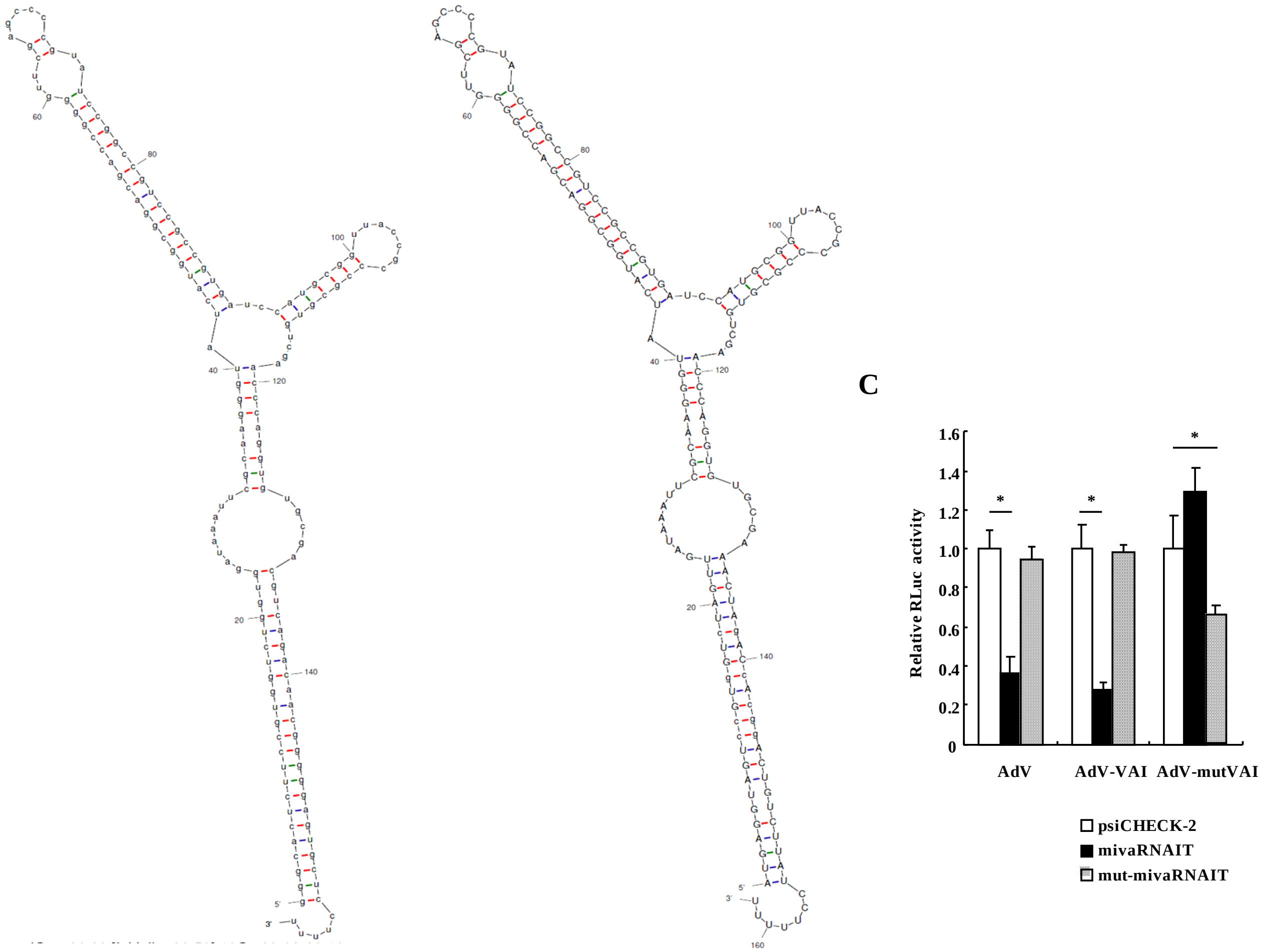

Figure S8

Suppression of the target gene expression by mivaRNAI and mutated mivaRNAI derived from AdV, AdV-VAI, and AdV-mutVAI.

(A, B) Mfold-predicted secondary structures of wild-type VA-RNA I (wt-VAI) (A) and mutated VA-RNA I (mut-VAI) (B). (C) HEK293 cells were transduced with AdV, AdV-VAI, and AdV-mutVAI and transfected with a control reporter plasmid (psiCHECK-2) or a reporter plasmid including two copies of mivaRNAI or mut-mivaRNAI complementary sequence in the 3'-UTR of the RLuc gene (mivaRNAIT or mut-mivaRNAIT). After 48 h incubation, luciferase activity was determined. The data show RLuc activity normalized by FLuc activity. The data are expressed as the means ± S.D. (n=4). *p<0.01.

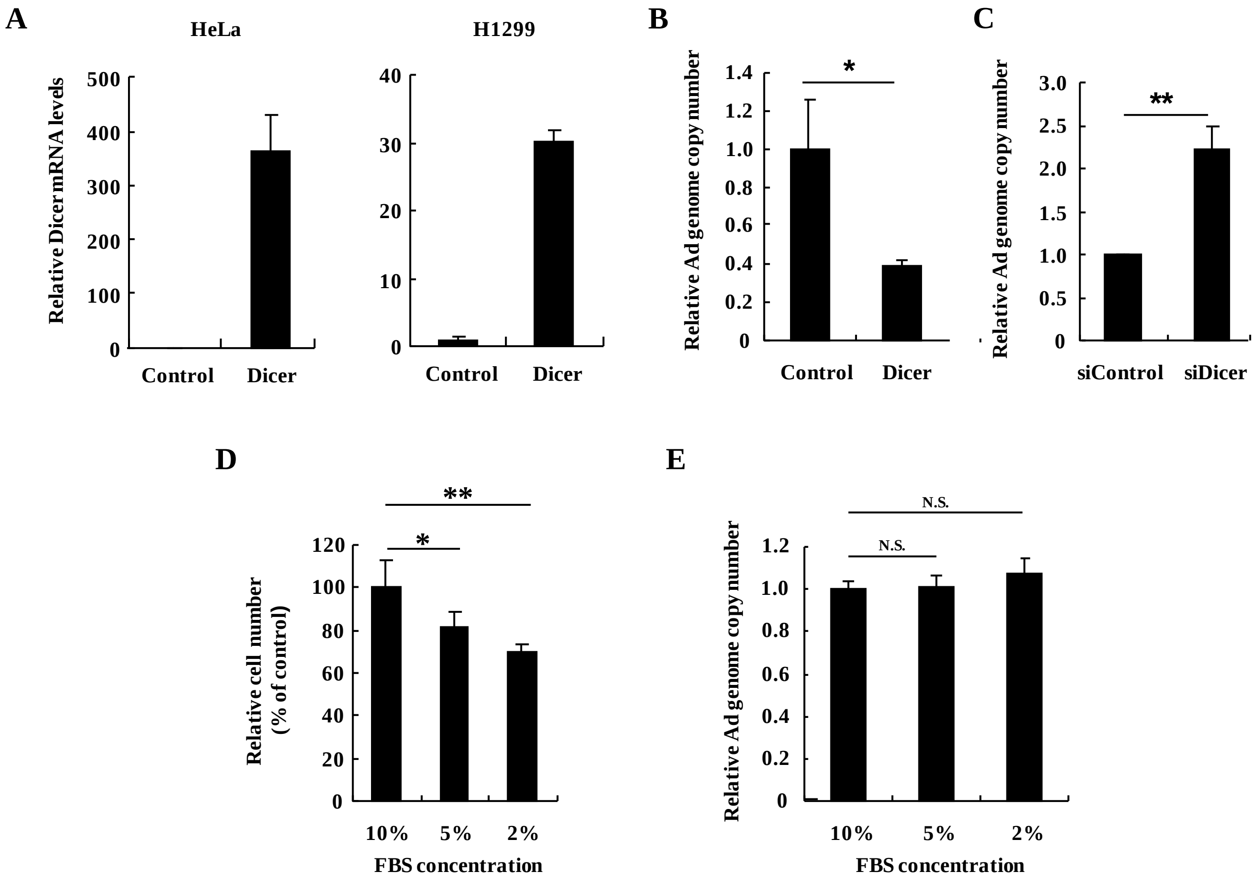

Figure S9

Dicer-mediated inhibition of Ad replication.

(A) HeLa and H1299 cells were transfected with a Dicer-expressing plasmid. After 48 h incubation, Dicer mRNA levels were determined by real-time RT-PCR analysis. Dicer mRNA levels in the cells transfected with a control plasmid were normalized to 1. (B, C) H1299 cells were transfected with a Dicer-expressing plasmid (p3XFLAG-CMV10-Dicer) (B) or siRNAs (C), followed by infection with WT-Ad at an MOI of 5. After 24 h incubation, copy numbers of WT-Ad genomic DNA in the cells were determined by real-time PCR analysis. (D) HeLa cells were cultured in the presence of FBS at the indicated concentrations. After 24 h incubation, the cell numbers were determined by Alamar blue assay. The data on cells cultured in 10% FBS-containing medium was normalized to 100%. (E) HeLa cells were cultured in the presence of FBS at the indicated concentrations for 24 h, followed by infection of WT-Ad. After 24 h incubation, the copy numbers of WT-Ad genomic DNA in the cells were determined by real-time PCR analysis. These data are expressed as the means ± S.D. (n=3-4). N.S.: not significantly different. *$p<0.05$, **$p<0.01$.

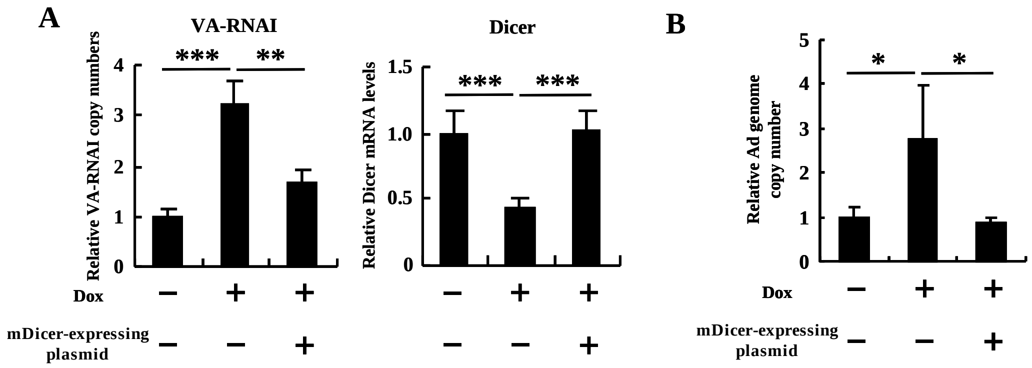

Figure S10

Canceling of shDicer-mediated upregulation of VA-RNA processing and Ad replication by silently-mutated Dicer (mDicer).

(A, B) HeLa-shDicer cells were transfected with p3XFLAG-CMV10-mDicer, a plasmid expressing a silently-mutated Dicer (mDicer) which is functional but resistant to shDicer, and were cultured in Dox-free or Dox-containing medium (100 ng/ml), followed by infection with WT-Ad at an MOI of 5. After 24 h incubation, the copy numbers of VA-RNAI (A) and WT-Ad genomic DNA (B) in the cells were determined by real-time RT-PCR and PCR analyses, respectively. These data are expressed as the means ± S.D. (n=4). *p<0.05, **p<0.01, ***p<0.001.

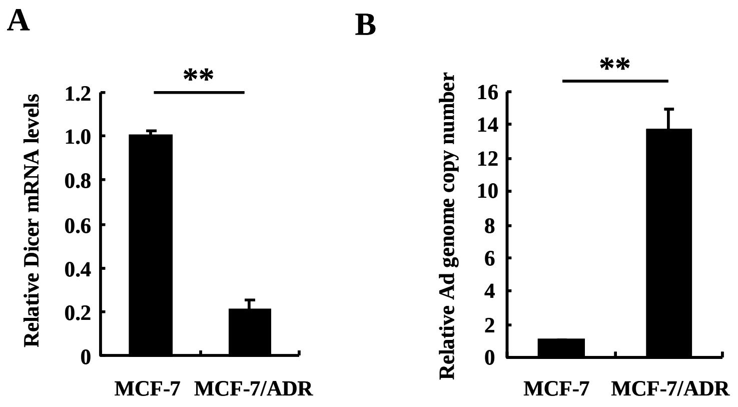

Figure S11

Levels of Dicer expression and WT-Ad replication in MCF-7/ADR cells.

(A) Dicer mRNA levels in MCF-7 and MCF-7/ADR cells were determined by real-time RT-PCR analysis. (B) MCF-7 and MCF-7/ADR cells were infected with WT-Ad. After 24 h incubation, copy numbers of WT-Ad genomic DNA were determined by real-time PCR analysis. The data are expressed as the means ± S.D. (n=3). **p<0.0001.

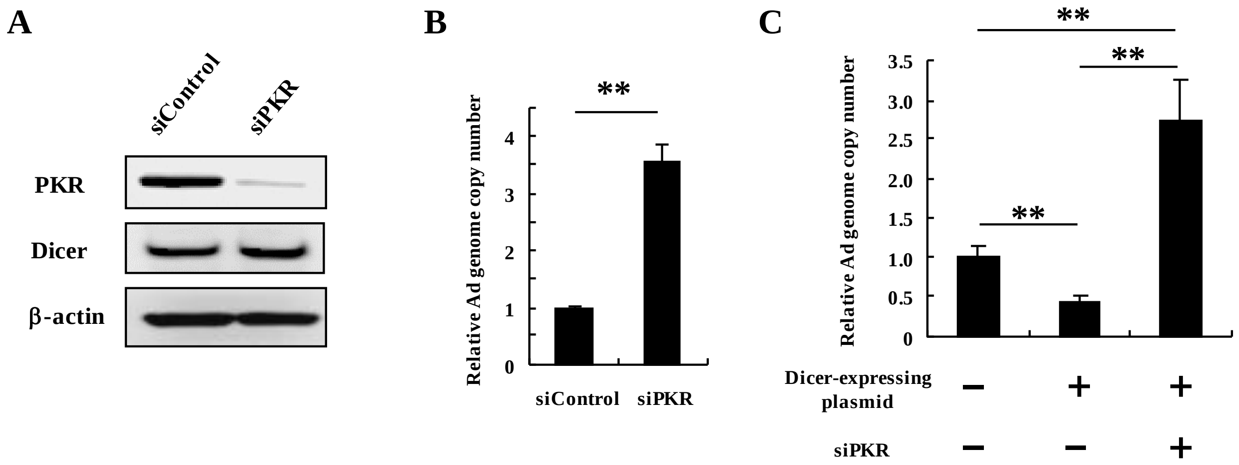

Figure S12

Involvement of PKR in Ad replication.

(A) HeLa cells were transfected with siControl or siPKR. After 48 h incubation, the PKR and Dicer protein levels were determined by western blotting analysis. (B) HeLa cells were transfected with siControl or siPKR, followed by infection with WT-Ad at an MOI of 5. After 24 h incubation, the copy numbers of WT-Ad genomic DNA in the cells were determined by real-time PCR analysis. (C) HeLa cells were co-transfected with siControl or siPKR and p3XFLAG-CMV10-Dicer, followed by infection with WT-Ad at an MOI of 5. After 24 h incubation, the copy numbers of WT-Ad genomic DNA were similarly determined. These data are expressed as the means ± S.D. (n=4). **p<0.001.

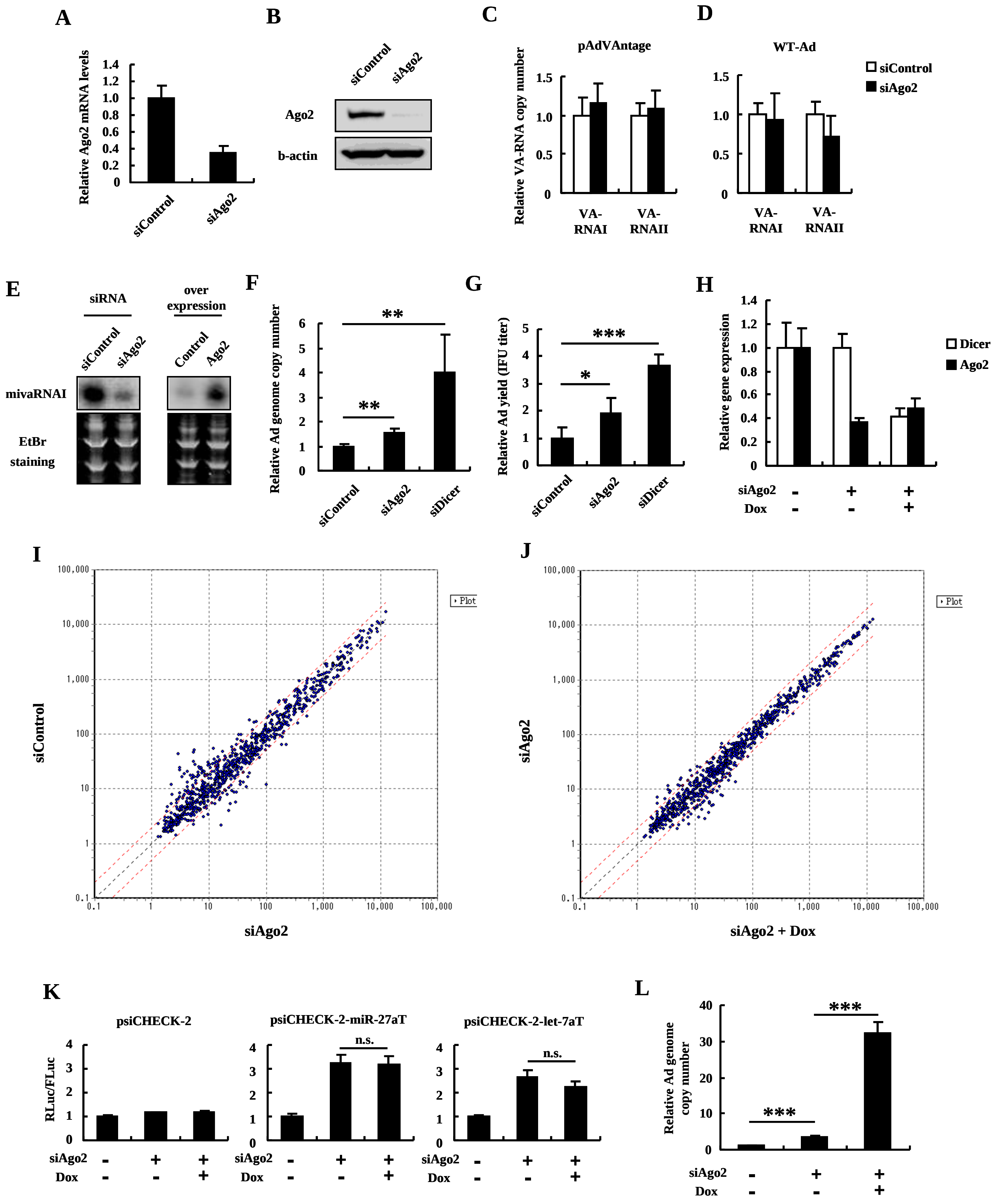
A
Relative Ago2 mRNA levels
siControl
siAgo2
B
siControl
siAgo2
Ago2
b-actin
C
pAdVAntage
Relative VA-RNA copy number
VA-RNAI
VA-RNAII
D
WT-Ad
siControl
siAgo2
E
siRNA
over expression
siControl
siAgo2
Control
Ago2
mivaRNAI
EtBr staining
F
Relative Ad genome copy number
siDicer
G
Relative Ad yield (IFU titer)
H
Relative gene expression
Dicer
Ago2
Dox
I
siAgo2
J
siAgo2 + Dox
K
psiCHECK-2
psiCHECK-2-miR-27aT
psiCHECK-2-let-7aT
RLuc/FLuc
n.s.
L
Relative Ad genome copy number

Figure S13

Involvement of Ago2 in VA-RNA processing and Ad replication.

(A, B) HeLa cells were transfected with siControl or siAgo2. After 48 h incubation, Ago2 mRNA (A) and protein (B) levels were determined by real-time RT-PCR analysis and western blotting analysis, respectively. Ago2 mRNA levels in the cells transfected with siControl were normalized to 1. (C) HeLa cells were co-transfected with siControl or siAgo2 and a VA-RNA-expressing plasmid (pAdVAntage). After 48 h incubation, the copy numbers of VA-RNAs were measured by real-time RT-PCR analysis. The copy numbers of VA-RNAs in the cells co-transfected with siControl and pAdVAntage were normalized to 1. (D) HeLa cells were transfected with siControl or siAgo2 and incubated for 48 h, followed by infection with WT-Ad. The copy numbers of VA-RNAs were similarly measured at 24 h after infection with WT-Ad. (E) siRNAs or an Ago2-expressing plasmid (p3XFLAG-CMV10-Ago2) were co-transfected with a VA-RNA-expressing plasmid (pAdVAntage) into HeLa cells. The expression levels of mivaRNAs were measured by northern blotting after 48 h incubation. The bottom panel of ethidium bromide (EtBr)-stained gels showed that comparable amounts of total RNA were loaded onto the gels. (F, G) HeLa cells were transfected with siRNAs, followed by infection with WT-Ad at an MOI of 5. After 24 h incubation, the copy numbers of WT-Ad genomic DNA (F) and IFU titers of progeny WT-Ad (G) in the cells were determined by real-time PCR analysis and infectious titer assay, respectively. (H-J) HeLa-shDicer cells were cultured in Dox-free or Dox-containing medium (100 ng/ml) for 48 h, followed by transfection with siRNAs. After 48 h incubation, Dicer and Ago2 mRNA levels (H) and miRNA expression profiles (I, J) in the cells were analyzed by real-time RT-PCR analysis and miRNA microarray analysis, respectively. The data of miRNA expression profiles were shown as scatter plots (I: a group without Dox and with siControl versus a group without Dox and with siAgo2; J: a group without Dox and with siAgo2 versus a group with Dox and siAgo2). (K) HeLa-shDicer cells were cultured in Dox-free or Dox-containing medium (100 ng/ml) for 48 h, followed by co-transfection with siRNAs and a control reporter plasmid (psiCHECK-2) or a reporter plasmid including two copies of miR-27a or let-7a complementary sequence in the 3'-UTR of the RLuc gene (psiCHECK-2-miR-27aT or -let-7aT). After 24 h incubation, luciferase activity was determined. The data show RLuc activity normalized by FLuc activity. (L) HeLa-shDicer cells were cultured in Dox-free or Dox-containing medium (100 ng/ml) for 48 h, followed by transfection with siRNAs. After 48 h incubation, the cells were infected with WT-Ad at an MOI of 5 for 24 h incubation, and the copy numbers of WT-Ad genomic DNA in the cells were determined by real-time PCR analysis. These data are expressed as the means ± S.D. (n=4). *<0.05, **p<0.01, ***p<0.001.

Table S1

Expression levels of highly expressed miRNAs in siAgo2-transfected HeLa-shDicer cells with and without Dox treatment.

No.	miRNA name	expression value		ratio
		Dox (-)	Dox (+)	
1	hsa-miR-23a-3p	12731.71	12809.63	1.006
2	hsa-let-7b-5p	11198.7	11480.44	1.025
3	hsa-miR-31-5p	9665.633	9325.029	0.965
4	hsa-miR-3960	8976.629	10144.6	1.130
5	hsa-miR-24-3p	8934.106	8773.455	0.982
6	hsa-miR-4497	8610.739	10477.68	1.217
7	hsa-miR-23b-3p	7303.661	7376.226	1.010
8	hsa-let-7c-5p	7007.318	6962.223	0.994
9	hsa-miR-3665	6542.424	7504.166	1.147
10	hsa-let-7a-5p	6526.671	6046.31	0.926
11	hsa-miR-92a-3p	6281.826	6731.325	1.072
12	hsa-miR-6869-5p	6020	5809.522	0.965
13	hsa-miR-6089	5779.271	6255.446	1.082
14	hsa-miR-6090	5775.1	5840.624	1.011
15	hsa-miR-222-3p	5184.862	4769.826	0.920
16	hsa-miR-26a-5p	4892.226	4479.428	0.916
17	hsa-miR-6087	4792.461	5291.427	1.104
18	hsa-miR-16-5p	4695.588	4015.729	0.855
19	hsa-miR-4787-5p	4545.784	5148.24	1.133
20	hsa-miR-221-3p	4539.04	4301.478	0.948
21	hsa-miR-7704	4449.627	5546.118	1.246
22	hsa-let-7i-5p	4247.616	3720.72	0.876
23	hsa-miR-17-5p	4027.939	3044.44	0.756
24	hsa-miR-20a-5p	3961.96	3258.24	0.822
25	hsa-miR-106a-5p	3742.573	2918.16	0.780
26	hsa-let-7e-5p	3173.871	3145.842	0.991
27	hsa-miR-6125	3165.038	3630.829	1.147
28	hsa-miR-8069	3138.005	3681.427	1.173
29	hsa-miR-125b-5p	3059.655	2979.535	0.974
30	hsa-miR-125a-5p	3051.026	2684.346	0.880
31	hsa-let-7d-5p	2877.605	2681.174	0.932
32	hsa-miR-7847-3p	2816.776	2920.964	1.037
33	hsa-miR-191-5p	2703.164	2584.027	0.956

Table S2

The oligonucleotides and primers used in this study

No.	name	sequence
1	mivaRNAIT-S	tcgagaaaaggagcactcccccgttgtctcagcaaaaggagcactccccgttgtctttaattaagcgc
2	mivaRNAIT-AS	ggccgcgcttaattaaagacaacgggggagtgctccttttgctgagacaacgggggagtgctcctttttc
3	mivaRNAIIT-S	tcgagaaggggctcgtccctgtttccggacagcaaggggctcgtccctgtttccggattaattaagcgc
4	mivaRNAIIT-AS	ggccgcgcttaattaatccggaaacagggacgagccccttgctgtccggaaacagggacgagccccttc
5	mut-mivaRNAIT-S	tcgagaactagaccacggactacctcacagcaactagaccacggactacctcattaattaagcgc
6	mut-mivaRNAIT-AS	ggccgcgcttaattaatgaggtagtccgtggtctagttgctgtgaggtagtccgtggtctagttc
7	shLuc-S	gatcccacgctgagtacttcgaaatttcaagagaatttcgaagtactcagcgtttttttggaaat
8	shLuc-AS	ctagatttccaaaaaacgctgagtacttcgaaattctcttgaaatttcgaagtactcagcgtgg
9	shDicer-S	gatcccgaatcagcctcgcaacaaattcaagagatttgttgcgaggctgattctttttggaaat
10	shDicer-AS	ctagatttccaaaaagaatcagcctcgcaacaaatctcttgaatttgttgcgaggctgattcgg
11	T7-VAΔmivaI-F	taatacgactcactatgggataaattcgcaagggta
12	T7-VAΔmivaI-R	gaagtcgcacacctgggttc
13	VA-RNAI-F	gggcactcttccgtggtctg
14	VA-RNAI-R	aggagcactcccccgttgtc
15	VA-RNAII-F	ggctcgctccctgtagccgg
16	VA-RNAII-R	aggggctcgtccctgtttcc
17	mut-VAI-F	atgaggtagtccgtggtcta
18	mut-VAI-R	aggataagacagtccgtggt
19	Ad5-F	gggatcgtctacctccttttga
20	Ad5-R	gggcagcagcggatgat
21	Ad5-probe	FAM-acagaaacccgcgctaccatactggag-TAMRA
22	GAPDH-F	ggtggtctcctctgacttcaaca
23	GAPDH-R	gtggtcgttgagggcaatg
24	GAPDH-probe	FAM-cactcctccacctttgacgctggg-TAMRA
25	Ad31-F	attgatgtggagtctgccgg
26	Ad31-R	acagggggctccggtaatat
27	Ad11-F	gcactgctatgaagacgggt
28	Ad11-R	tccgggcaatccaactgaaa
29	Ad35-F	tccgtggactgtgatttgca
30	Ad35-R	ccaacattggcagccttcac
31	Ad4-F	agacagcgactcttcactgc
32	Ad4-R	tcgtcctcatcatcgcttgg
33	Dicer-F	gggagacttcagcagaaatggaa
34	Dicer-R	ctgacagctgacacttgttgagca
35	Ago2-F	cgcgtccgaaggctgctcta
36	Ago2-R	tggctgtgccttgtaaaacgct
37	mDicer-F	ggacatggcattgggaaaaaccaaccgcggaataagcagatggaagcagaattcag
38	mDicer-R	ctgaattctgcttccatctgcttattccgcggttggtttttcccaatgccatgtcc

39	miR-27aT-S	tcgaggcggaacttagccactgtgaacagcgcggaacttagccactgtgaattaattaagcgc
40	miR-27aT-AS	ggccgcgcttaattaattcacagtggctaagttccgcgctgttcacagtggctaagttccgcc
41	let-7aT-S	tcgagaactatacaacctactacctcacagcaactatacaacctactactcattaattaagcgc
42	let-7aT-AS	ggccgcgcttaattaatgaggtagtaggttgtatagttgctgtgaggtataggttgtatagttc

Table S3

The primary antibodies used in this study

No.	antigen	type	Company
1	p-eIF2α	rabbit	Cell Signaling Technology, Danvers, MA
2	eIF2α	rabbit	Cell Signaling Technology, Danvers, MA
3	Hexon	mouse	Abnova, Taipei, Taiwan
4	Fiber	mouse	Abcam, Cambridge, UK
5	β-actin	mouse	Sigma-Aldrich Japan, Tokyo, Japan
6	Dicer	mouse	Abcam, Cambridge, UK
7	PKR	mouse	Santa Cruz Biotechnology, Santa Cruz, CA
8	Ago2	mouse	Wako, Osaka, Japan
